# Hepatitis C elimination in Myanmar: Modelling the impact, cost, cost-effectiveness and economic benefits

**DOI:** 10.1016/j.lanwpc.2021.100129

**Published:** 2021-03-23

**Authors:** Nick Scott, Thin Mar Win, Tom Tidhar, Hla Htay, Bridget Draper, Phyo Thu Zar Aung, Yinzong Xiao, Anna Bowring, Christian Kuschel, Sonjelle Shilton, Khin Pyone Kyi, Win Naing, Khin Sanda Aung, Margaret Hellard

**Affiliations:** a Burnet Institute Melbourne, 85 Commercial Rd, Melbourne 3004, Victoria, Australia; b School of Public Health and Preventive Medicine, Monash University, 553St Kilda Rd, Melbourne 3004, Victoria, Australia; c Burnet Institute Myanmar, Second floor, 226U Wisara Road, Wizaaya Plaza, Bahan Township, Yangon, Myanmar; d University of Melbourne, Parkville 3010, Victoria, Australia; e Foundation for Innovative New Diagnostics (FIND), Yangon, Myanmar; f Myanmar Liver Foundation, 33-35, First Floor, Pathein Street, KyunTaw (Middle) Ward, Sanchaung Township, Yangon, Myanmar; g Department of Hepatology, 500 bedded Specialty Hospital, University of Medicine, Yangon, Myanmar; h National Hepatitis Control Program, Myanmar Ministry of Health, Myanmar; i Department of Infectious Diseases, The Alfred and Monash University, Melbourne 3004, Victoria, Australia; j The Peter Doherty Institute for Infection and Immunity, 792 Elizabeth St, Melbourne 3000, Victoria, Australia

**Keywords:** Elimination, Hepatitis C, Low and middle income country, Mathematical model, Myanmar

## Abstract

**Background:**

Myanmar has set national hepatitis C (HCV) targets to achieve 50% of people diagnosed and 50% treated by 2030. The WHO has additional targets of reducing incidence by 80% and mortality by 65% by 2030. We aimed to estimate the impact, cost, cost-effectiveness and net economic benefit of achieving these targets.

**Methods:**

Mathematical models of HCV transmission, disease progression and the care cascade were calibrated to 15 administrative regions of Myanmar. Cost data were collected from a community testing and treatment program in Yangon. Three scenarios were projected for 2020–2030: (1) baseline (current levels of testing/treatment); and testing/treatment scaled up sufficiently to reach (2) the national strategy targets; and (3) the WHO targets.

**Findings:**

Without treatment scale-up, 333,000 new HCV infections and 97,000 HCV-related deaths were estimated to occur in Myanmar 2020–2030, with HCV costing a total $100 million in direct costs (testing, treatment, disease management) and $10.4 billion in lost productivity. In the model, treating 55,000 people each year was sufficient to reach the national strategy targets and prevented a cumulative 40,000 new infections (12%) and 25,000 HCV-related deaths (25%) 2020–2030. This was estimated to cost a total $189 million in direct costs ($243 per DALY averted compared to no treatment scale-up), but only $9.8 billion in lost productivity, making it cost-saving from a societal perspective by 2024 with an estimated net economic benefit of $553 million by 2030. Reaching the WHO targets required further treatment scale-up and additional direct costs but resulted in greater longer-term benefits.

**Interpretation:**

Current levels of HCV testing and treatment in Myanmar are insufficient to reach the national strategy targets. Scaling up HCV testing and treatment in Myanmar to reach the national strategy targets is estimated to generate significant health and economic benefits.

**Funding:**

Gilead Sciences.

**Research in Context**

*Evidence before this study*

Hepatitis C (HCV) testing and treatment has been found to be highly cost-effective in low- and middle-income countries (LMICs), however the key issue for LMICs is the affordability of a public health response to hepatitis C rather than the cost-effectiveness of testing and treatment. A major evidence gap preventing progress towards elimination is that governments are unlikely to implement large-scale HCV programs without baseline estimates of the health and economic burden of HCV, as well as estimates of the total investment requirements and expected health and economic benefits of elimination.

In Myanmar, a national action plan and monitoring and evaluation framework have been developed, and clinical guidelines are being regularly updated. However, to support the financing of a large-scale HCV elimination program, evidence is required for the investment requirements and health and economic benefits of elimination.

*Added value of this study*

In this study, we use economic modelling, based on primary costing data collected from community-based testing and treatment programs, to estimate the investment requirements and health and economic benefits of HCV elimination in Myanmar.

The study found that to reach the Myanmar national strategy targets of 50% of people with HCV diagnosed and 50% treated by 2030, HCV treatment should be scaled up from an estimated 4000 per year to approximately 55,000 per year, which could avert 40,000 new HCV infections and 25,000 HCV-related deaths between 2020 and 2030. When the full set of economic benefits are considered, scaling up testing and treatment to achieve the national strategy targets is likely to become cost saving by 2024 and produce a net economic benefit of $553 million by 2030.

*Implications of all the available evidence*

Scaling up HCV testing and treatment in Myanmar to reach the national strategy targets is estimated to generate significant health and economic benefits. The findings of this study are being used to support the financing of larger scale hepatitis C elimination strategy, and to assist decision-makers in Myanmar on the prioritisation of funds between hepatitis C and other health issues.

Alt-text: Unlabelled box

## 1 Introduction

Hepatitis C virus (HCV) is a blood-borne infection transmitted through injecting drug use, unsafe medical procedures, and other community exposures [1]. Globally, over 70 million people are infected with HCV [2] and close to 400,000 people die annually due to HCV-related conditions [1], disproportionately in low- and middle-income countries (LMICs) [3]. The recent major advancement of highly effective (>90%), short duration (8–12 weeks) and highly tolerable direct-acting antiviral (DAA) treatment regimens for HCV [4,5] is a game changer with the potential to significantly reduce HCV-related morbidity and mortality. The discovery of DAAs means that HCV elimination is now being viewed as a realistic public health goal [6].

The simplicity of DAA treatments is also revolutionising existing models of HCV care. HCV treatment can now be managed in primary healthcare and community settings rather than being confined to hospital settings as previously occurred. In addition, the availability of a range of novel point-of-care diagnostic tests [7,8] is further facilitating the expansion of HCV services by removing the reliance on centralised laboratories. Changes in the treatment landscape have resulted in the World Health Organization (WHO) developing its first Global Health Sector Strategy to eliminate hepatitis, setting targets to reduce HCV incidence by 80% and HCV-related mortality by 65% by 2030 [6]. Many countries have since developed their own HCV strategies.

In Myanmar, HCV antibody prevalence is estimated to be 2.7% [9], or almost 1.5 million people that have been exposed to HCV. Population-level studies of RNA prevalence have not been conducted, but with an estimated 26% spontaneous clearance rate for HCV this would suggest that at least 1.1 million people in Myanmar are living with chronic HCV. In 2017, the Department of Public Health in Myanmar released their first National Action Plan for Viral Hepatitis Response 2016–2020 [10], which set targets to achieve 50% of people with HCV diagnosed and 50% of people with HCV treated by 2030. Alongside the release of this strategy, the public sector HCV treatment program was launched, which had treated approximately 2000 people (achieved a sustained viral response after 12 weeks [SVR-12]) across nine hospitals throughout the country by the end of December 2018, and has since been expanded.

For Myanmar to reach either the national strategy targets or the WHO targets, investment in prevention, testing and treatment of HCV will be required. In addition to high rates of infection and transmission among people who inject drugs (PWID) in Myanmar, data indicate that transmission is also occurring within the formal and non-formal health sector, as is observed in other LMICs [11], [12], [13]. This means that as well as increased harm reduction among PWID, additional prevention activities targeting behaviour change [12] will be required to reduce incidence.

Throughout 2019–20, the Burnet Institute partnered with the Foundation for Innovative New Diagnostics (FIND) in a UNITAID funded study in Yangon, Myanmar (the “Hepatitis C: community testing and treatment (CT2)” study) to assess the feasibility of HCV point-of-care testing for antibody and RNA, with the provision of treatment for those patients identified as HCV RNA positive [14]. Between 30 January and 30 September 2019, 633 participants were recruited from two community-based clinics, one clinic focusing on the general population infected with HCV run by Myanmar Liver Foundation and a second clinic with a focus on PWID run by Burnet Institute [14]. Follow-up of participants was conducted up to 31 August 2020. Of the 633 participants, 606/633 were HCV antibody positive and reflexively tested for HCV RNA using a GeneXpert HCV RNA test [15], 535/606 were HCV RNA positive, 489/535 met the study eligibility criteria for treatment (individuals who had previously been treated for HCV, who were co-infected with HIV, hepatitis B or tuberculosis, who had renal impairment or who were pregnant were not eligible for treatment through the study), 488/489 started treatment, and 484/488 completed treatment [16]. The CT2 study provides valuable costing data on a possible model of testing and treatment in Myanmar, which can be combined with epidemiological and behavioural data from the cross-sectional seroprevalence survey among the general population (from 2015; 18 sites, *n* = 55,47 [9]) and two cross-sectional Integrated Biological and Behavioural Surveillance Survey (IBBS) among PWID (from 2014: 10 sites, *n* = 3340; [17] and from 2017–2018: 13 sites, *n* = 6061 [18]) to assess the affordability and potential economic benefits of scaling up testing and treatment interventions for HCV.

This study uses mathematical and economic modelling, informed by the CT2 study, to determine the testing and treatment scale-up required for Myanmar to achieve the 2030 National Strategy diagnosis and treatment targets and the 2030 WHO incidence and mortality targets. We estimate the total cost, cost-effectiveness and net economic benefit of achieving these targets. The modelling was performed independently for each of the 15 states, regions and union territories of Myanmar, with the regional models aggregated to form national estimates. In Myanmar, an increasing number of people are being treated through an increasing number of sites across the country, a national action plan and monitoring and evaluation framework have been developed, and clinical guidelines are being regularly updated; however findings of this study are required to catalyse the financing needed to make HCV treatment a reality for people in Myanmar.

## 2 Methods

### 2.1 Setting

Myanmar has an estimated population of 52 million and HCV antibody prevalence of 2.7% [9] (i.e. almost 1.5 million people have been exposed to HCV). The HCV antibody prevalence in key risk populations, such as the estimated 75,000 PWID [19], is as high as 80% in some regions [20,21]. However, this accounts for a modest proportion of the 1.5 million exposed individuals, suggesting that other risk factors such as unsafe medical practices drives HCV transmission [9]. In addition, approximately 50% of HCV-infected PWID in Myanmar are co-infected with HIV [22] an important co-morbidity that can significantly change the outcomes of HCV infection [23], however studies on co-infection in Myanmar are smaller and data is more limited.

### 2.2 Model description

**Fig. 1:**
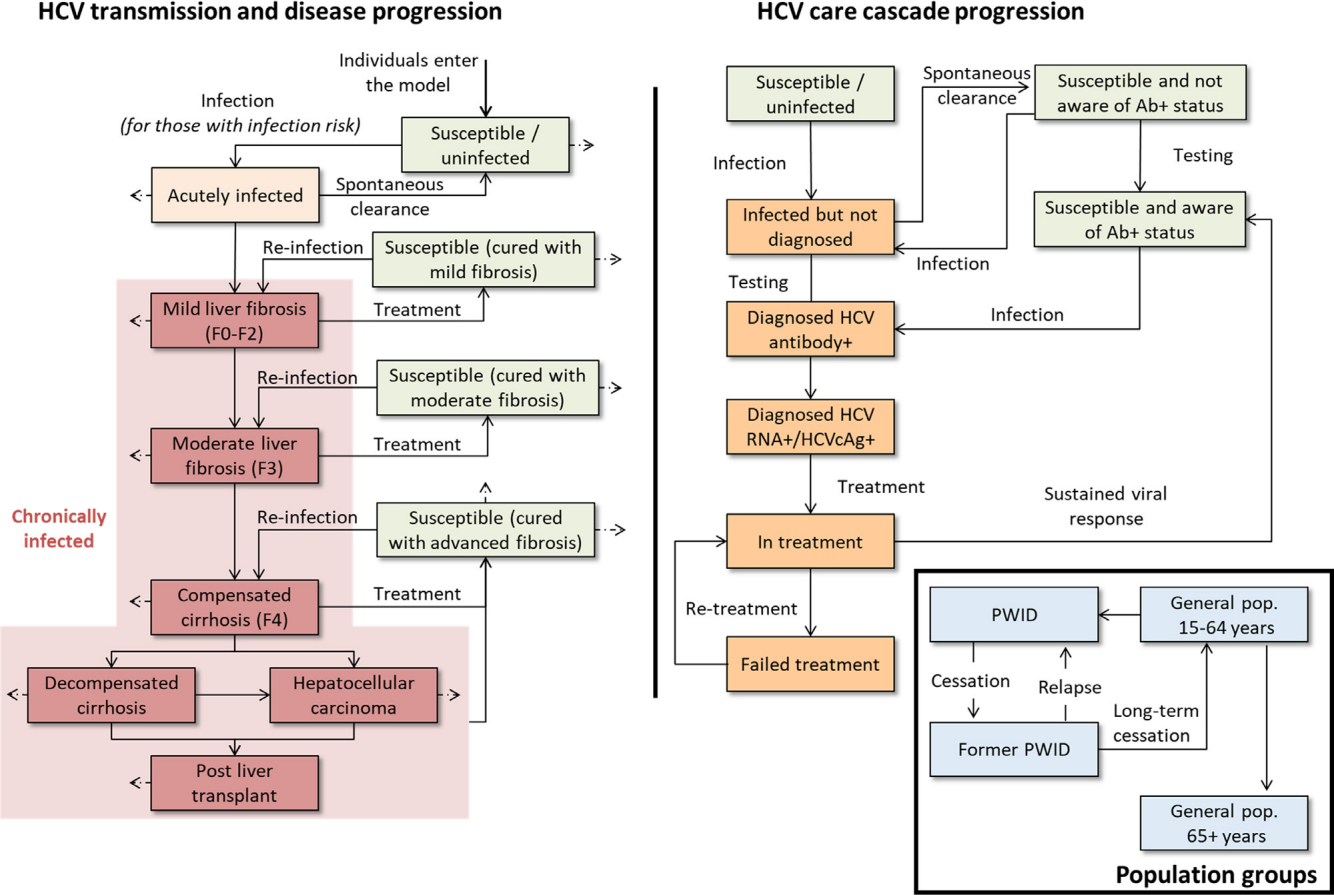
**Hepatitis C transmission, disease progression and care cascade model schematic**. Not shown for brevity: liver fibrosis stages F0, F1 and F2 were all explicitly modelled; acute stage of infection and spontaneous clearance was possible after re-infection as well as primary infection.

An epidemic model of HCV transmission, disease progression and the cascade of care (Fig. 1) was developed for each of the 15 geographical divisions (states, regions and union territories) of Myanmar based on previous work [24,25]. In brief, each sub-national model tracks the population according to:

• population group (general population 15–64 years, general population 65+ years, PWID, former PWID—separated due to different infection risks, prevalence and interventions);
• infection status (susceptible [naïve or previously cured], acutely infected, and chronically infected);
• stage of liver disease; and
• progression through the HCV care cascade (undiagnosed, diagnosed HCV antibody positive, diagnosed HCV RNA positive / HCVcAg positive, currently on treatment, or cured and exposed [i.e. antibody+/RNA–and aware of their Ab status]).

Transmission was modelled to occur among PWID as well as among the rest of the general population. Model parameters, summaries of input data and sources are provided in Table 1, and further model details are in Appendix A.

**Table 1:** Parameter estimates and data inputs for the HCV models.

Variables	Range	Sources
*HCV-related parameters*		
Spontaneous clearance	26%	Micallef et al. [27]
Duration of acute stage	12 weeks	Mondelli et al. [28]
Treatment effectiveness	95%	Lawitz et al. [4], Gane et al. [29,30], Poordad et al. [31]
*Annual transition probabilities*		
F0→F1	11.7% (10.4–13.0%)	Thein et al. [32] Reported values and ranges are global estimates, and so to apply to Myanmar context probabilities were scaled between these bounds such that the mortality over time in the model matches local estimates. The overall scaling factor from the calibration that was applied to these values was 0.96.
F1→F2	8.5% (7.5–9.6%)	
F2→F3	12.0% (10.9–13.3%)	
F3→F4	11.6% (10.4–12.9%)	
F4→DC	3.7% (3.0–9.2%)	National Centre in HIV Epidemiology and Clinical Research [33]. Probabilities were scaled between bounds to fit the mortality over time in Myanmar. The overall scaling factor from the calibration that was applied to these values was 0.96.
F4→HCC	1.0% (0.9%−3.8%)	
DC→HCC	6.8% (4.1–9.9%)	
DC→death	13.8% (7.4–20.2%)	
HCC→death	60.5% (54.5–67.6%)	
F4→DC (post cure)	74% reduced risk	Nahon et al. [34] hazard ratio = 0.26 (0.17–0.39)
DC→HCC (post cure)	71% reduced risk	Nahon et al. [34] hazard ratio = 0.29 (0.13–0.43)
DC→death (post cure)	73% reduced risk	Nahon et al. [34] hazard ratio = 0.27 (0.18–0.42) for overall mortality following SVR for patents with cirrhosis.
HCC→death (post cure)	73% reduced risk	
*Disutility weightings*		
F0–2	0.012	No GBD estimate for F0–4, so used disability weights from Martin et al. [35]: for F0–2 and F3–4 used value for mild and moderate abdominopelvic problem [36] respectively, assuming linear disability increase from mild chronic HCV to compensated cirrhosis (in line with other estimates [37]).
F3–4	0.068	
DC	0.194	Disability weights used in the Global Burden of Disease study [38]
HCC	0.508	
Average years of life lost from an HCV-related death	18.35 years	Used to calculate the years of life lost component of DALYs, based on remaining life expectancy from WHO Global Health Observatory data. Calculated assuming HCV-related deaths are age-distributed as described in the below table entry, and using the 2019 expectation of life at age x for both sexes in Myanmar [39]
*Other parameters*		
Discounting	3% per annum	Applied to both costs and DALYs
Additional injecting-related mortality	0.0235 per year	Mathers et al. systematic review [40]
*National characteristics*		
15–64 year old population size	Time varying. At start of 2014: 32,982,768	Myanmar Information Management Unit [41]
65+ year old population size	Time varying. At start of 2014: 2897,563	Myanmar Information Management Unit [41]
PWID population size	Time varying. At start of 2018: 93,215, range 49,677 – 124,287	Myanmar Integrated Biological and Behavioural Surveillance Survey among PWIDs (2014 and 2017–2018) [17],[18]
Total people with HCV	Time varying. At start of 2015: 1002,787	Total people living with HCV = HCV antibody prevalence in 2015 [9] * 74% (spontaneous clearance rate) * national population in 2015.
HCV-related mortality	Time varying. At start of 2016: 8760	HCV-related liver cancer deaths was sourced from 2017 Global Burden of Disease study [42]
HCV Ab+ prevalence among PWID	Time varying. At start of 2018: 56%	Myanmar Integrated Biological and Behavioural Surveillance Survey among PWIDs (2014 and 2017–2018) [17,18]
HCV Ab+ prevalence in general population	Time varying. At start of 2015: 2.7%	A cross-sectional sero-survey in 2015 [9]
Incidence of HCV	Time varying. At start of 2016: 46,848	Global Burden of Disease 2017 [42]
People in each liver disease stage	For 2016: F0–2, 78% F3+, 22%	These were outcomes of the calibration, where progression rates were varied to produce the estimated mortality. This compares to estimates of ~30% from the 2017–18 public sector HCV treatment program and ~6% from the CT2 study (both with differently biased populations).
Proportion of people living with HCV who are diagnosed	In 2015: <1%	There is a lack of HCV notification data and no accurate diagnosed proportion data available in Myanmar. Myanmar National Action Plan for Viral Hepatitis Response 2017–2020 [10]
*Costs*		
Testing: Antibody negative patients	$16.88	CT2 costing study (Appendix C). Includes $1.17 for Ab test as well as $5.27 staff costs and $11.33 fractional overhead costs.
Testing: Antibody positive / RNA negative patients	$51.39	CT2 costing study (Appendix C). Includes antibody test + $34.51 for RNA test (reflexive).
Testing: Antibody positive / RNA positive patients	$51.39	As per antibody positive / RNA negative patients as same procedure is involved.
Treatment	$207.84	CT2 costing study (Appendix C). Costs are in addition to diagnostic testing. Based on 12-week course, includes drug cost of US$86.76 as well as $4.21 staff costs, $34.51 for an SVR12 RNA test and $45.32 fractional overhead costs (across 4 visits).
Disease management		Cost estimates for F3, F4, DC, and HCC from hepC calculator tool [43]. F0–2 assumed to be $0 based on lack of available services. Disease management costs were only applied to 9% of people (expert opinion). Sensitivity analysis used to test when 25% of people incurred disease management costs.
*F0–2*	$0	
*F3*	$160	
*F4*	$187	
*DC*	$2075	
*HCC*	$3815	
Cost per year of productive life lost	$1196	Per capita GDP [44]
*Productivity parameters*		
Employment rate		
*General population*	65%	The World Bank [45]
*PWID*	62%	CT2 study
Per capita gross domestic product	US$1196	The World Bank [44]
Lost productivity attributable to hepatitis C		
*Absenteeism*	1.85%	Dibonaventura et al. [46] US study. People with hepatitis C had 4.88% absenteeism versus 3.03% for people without hepatitis C.
*Presenteeism*	3.19%	Dibonaventura et al. [46] US study. People with hepatitis C had 16.69% presenteeism versus 13.50% for people without hepatitis C.
Additional productivity losses for people with cirrhosis		
*Absenteeism*	2.79 times	Younossi et al. [47]; European study.
*Presenteeism*	1.54 times	
Relative reduction in absenteeism following hepatitis C cure		
*Cirrhotic*	44%	Younossi et al. [47]
*Non-cirrhotic*	0%	
Relative reduction in presenteeism following hepatitis C cure		
*Cirrhotic*	11%	Younossi et al. [47]
*Non-cirrhotic*	20%	
Percentage of hepatitis C-related deaths occurring at different age brackets in 2016		WHO 2016 estimates for Myanmar [48]
*15–29*	1%	
*30–49*	16%	
*50–59*	23%	
*60+*	60%	

Co-infection with HIV was not included explicitly, but rates of disease progression were calibrated to account for the fact that among people with HCV, approximately 50% are coinfected with HIV [22,26] and approximately 20% are coinfected with HBV [26], and that those with coinfections could be expected to develop liver disease at increased rates [23].

### 2.3 Epidemiological data

Population sizes (and growth) for each region were estimated from the Myanmar Information Management Unit for the general population and from the IBBS [17,18] for PWID.

Estimates of HCV prevalence among PWID were taken from the IBBS [17,18], and among the general population from the 2015 cross-sectional seroprevalence survey [9]. For geographical regions where estimates were missing, population-weighted averages from available regions were used to generate national averages, which were then applied.

National estimates of HCV incidence and HCV-related mortality were taken from the Global Burden of Disease study [42], which were divided among regions based on the estimated number of people with HCV.

National epidemiological data (aggregated across the regions) are provided in Table 1 with further calculation and source details and region-specific data in the supplement (Appendix B).

### 2.4 Costs

The economic costs of testing, treatment, disease management and lost productivity were calculated for each scenario, following the methods of a recent global investment framework [49]. Costs are presented in 2018 US$ (based on an exchange rate of 1500Kyats per USD in the second half of the year, when the study was funded) and discounted at 3% per annum. Results are reported from a health systems perspective (only considering testing, treatment and disease management costs) as well as from a societal perspective (additionally considering the costs of lost productivity).

Cost estimates for providing testing and treatment services were collected from the CT2 study sites using a micro-costing approach. The average cost per person to provide antibody testing, RNA testing and treatment services were calculated by identifying and assigning a cost all consumables (e.g. drugs, medical supplies, diagnostic tests as well as auxiliary tests such as liver functions tests), resources (e.g. staff time, recorded as minutes of healthcare worker, administration and lab technician time) and overheads (e.g. a proportion of the cost of utilities, phones, computers and other equipment) used to deliver the service (Appendix C).

In order to cost a scaled-up testing program, a test positivity rate estimate is required. For PWID, it was based on prevalence (i.e. in a 50% prevalence risk group it would require on average two tests to obtain one positive), while for the general population, testing was assumed to be partly targeted and conducted twice as well as random selection (i.e. if the general population prevalence was approximately 1%, this implies that it would require 50 tests to obtain one positive result). It was assumed that three out of four HCV RNA tests among people diagnosed antibody positive resulted in one positive result, based on approximately a 25% spontaneous clearance rate. Following cure or Ab+/RNA- diagnosis, RNA tests were used for re-screening and positivity rates were decreased accordingly.

Disease management costs were only considered for people with HCV who are likely to utilize health services as a result of their infection. It was estimated that no one incurred disease costs for F0–2 liver disease stages due to lack of available services, and that 9% of diagnosed or cured people with F3, F4, decompensated cirrhosis (DC) or hepatocellular carcinoma (HCC) liver disease stages, as well as 9% of undiagnosed people DC or HCC, are likely to access health services for these conditions [50] (sensitivity analysis tested the impact if this was 25% or 50%).

The cost of lost productivity due to absenteeism (HCV-related sick days), presenteeism (people being less productive as a result of their illness) and premature deaths were calculated using the human capital approach [49,51]. Years of potential productive life lost among people with HCV before and after cure were calculated by multiplying estimated rates of absenteeism and presenteeism [46] by the employment rate, with different rates of absenteeism and presenteeism applied for people with/without cirrhosis and pre/post cure [47], and a reduced employment rate used for PWID compared to the general population. Years of potential productive life lost due to premature deaths were calculated by dynamically tracking a population of people who died from HCV from their age at death, based on WHO estimates for Myanmar [48] for the age-distribution of HCV-related deaths, until the assumed retirement age of 60 years. Years of potential productive life lost were converted to economic outcomes using per capita gross domestic product. Additional details are provided in Appendix A.

### 2.5 Model calibration

For each region, the model was calibrated to time series data on the prevalence of HCV among the general population, the prevalence of HCV among PWID, the annual number of HCV-related deaths, the total number of people living with HCV, the estimated incidence of HCV, and the proportion of people living with HCV who were diagnosed (Table S1). This involved simultaneously varying parameters for: the force of infection among PWID (the force of infection was dynamic and dependent on prevalence, but a constant scalar factor was varied), the average length of injecting career among PWID, the disease progression rates (F0→F1, F1→F2, F3→F4, F4→DC, F4→HCC, DC→HCC), the annual probability of dying from DC, the annual probability of dying from HCC, and the annual probability of having a HCV antibody test for each population group.

The calibrated model fits for each region are provided with the sub-national projections and results in Appendix D.

### 2.6 Scenarios

Three scenarios were projected from 2020 to 2030 for each region:

1 *Baseline (status-quo)*: This scenario includes the treatment scale-up that has occurred so far (approximately 4000 courses per annum in the public and non-governmental organisation sectors since 2017 [52]), with treatment numbers maintained at this level up to 2030.
2 *National strategy*: Annual testing and treatment numbers in the model for the period 2020–2030 increased such that they were sufficient to reach the national strategy targets (50% diagnosed and 50% treated by 2030)
3 *WHO targets*: Annual testing and treatment numbers in the model for the period 2020–2030 increased such that they were sufficient to reach the WHO targets (80% reduction in incidence and 65% reduction in mortality in 2030 compared with 2015).

Outputs from an additional counterfactual scenario of no treatment are provided in Appendix E.

### 2.7 Outcomes

Outcomes considered for each region and scenario were the projected number of people with HCV, HCV incidence, HCV-related deaths, HCV prevalence among the general population and among PWID, HCV-related disability-adjusted life years (DALYs) and costs (testing, treatment, disease management and lost productivity). DALYs and costs were discounted at 3% per annum.

At a national level (by aggregating the sub-national projections), the incremental cost-effectiveness ratio (ICER; difference in costs / difference in DALYs) was calculated for the national strategy and WHO strategy compared to the baseline from a health systems perspective.

At a national level, the net economic benefit over time (the difference in total cumulative costs [49] compared to the baseline) was calculated from a societal perspective.

### 2.8 Uncertainty analysis

A multivariate probabilistic uncertainty analysis was conducted to estimate 95% credible intervals (95%CrI) for outcomes. Model projections were run 100 times with model parameters (from Table 1, HCV parameters, direct costs, health utilities and productivity loss parameters) drawn at random from uniform distributions between their individual uncertainty bounds or +/−10% their point estimates. The central 95 percentiles of outputs are reported as 95%CrIs.

### 2.9 Sensitivity analysis

One-way sensitivity analyses were undertaken to determine the effect on outcomes if: the test positivity rate was halved; 25% or 50% of people incurred liver disease management costs rather than 9%; 0% or 100% of staffing and overhead costs were included rather than 50%; the price of DAAs was $200 or $40 rather than $86.76; baseline incidence was 50% higher or 50% lower than reported; and baseline mortality was 50% higher or 50% lower than reported.

In addition, theoretical scenarios were run where interventions to improve primary prevention among the general population were implemented over the period 2020–2025 and maintained to 2030, such that by 2025 a 20%, 40% or 60% reduction in the force of infection among the general population had been achieved. Similarly, scenarios were run where harm reduction interventions among PWID were scaled up over the period 2020–2025 and maintained to 2030 such that by 2025 a 20%, 40% or 60% reduction in the force of infection among PWID had been achieved.

### 2.10 Ethics

Ethics for the CT2 study and this modelling study were obtained from the Alfred Health ethics committee in Australia (Alfred HREC reference 244/17 and 327/18 respectively) and the department of medical research (DMR) Institutional Review Board (DMR IRB, formerly known as ethics review committee (DMR ERC)) in Myanmar (DMR/2018/144 and DMR/2019/141 respectively).

### 2.11 Role of the funding source

The funders (Gilead Sciences) had no role in study design, data collection and analysis, or decision to publish.

## 3 Results

### 3.1 Baseline scenario

**Fig. 2:**
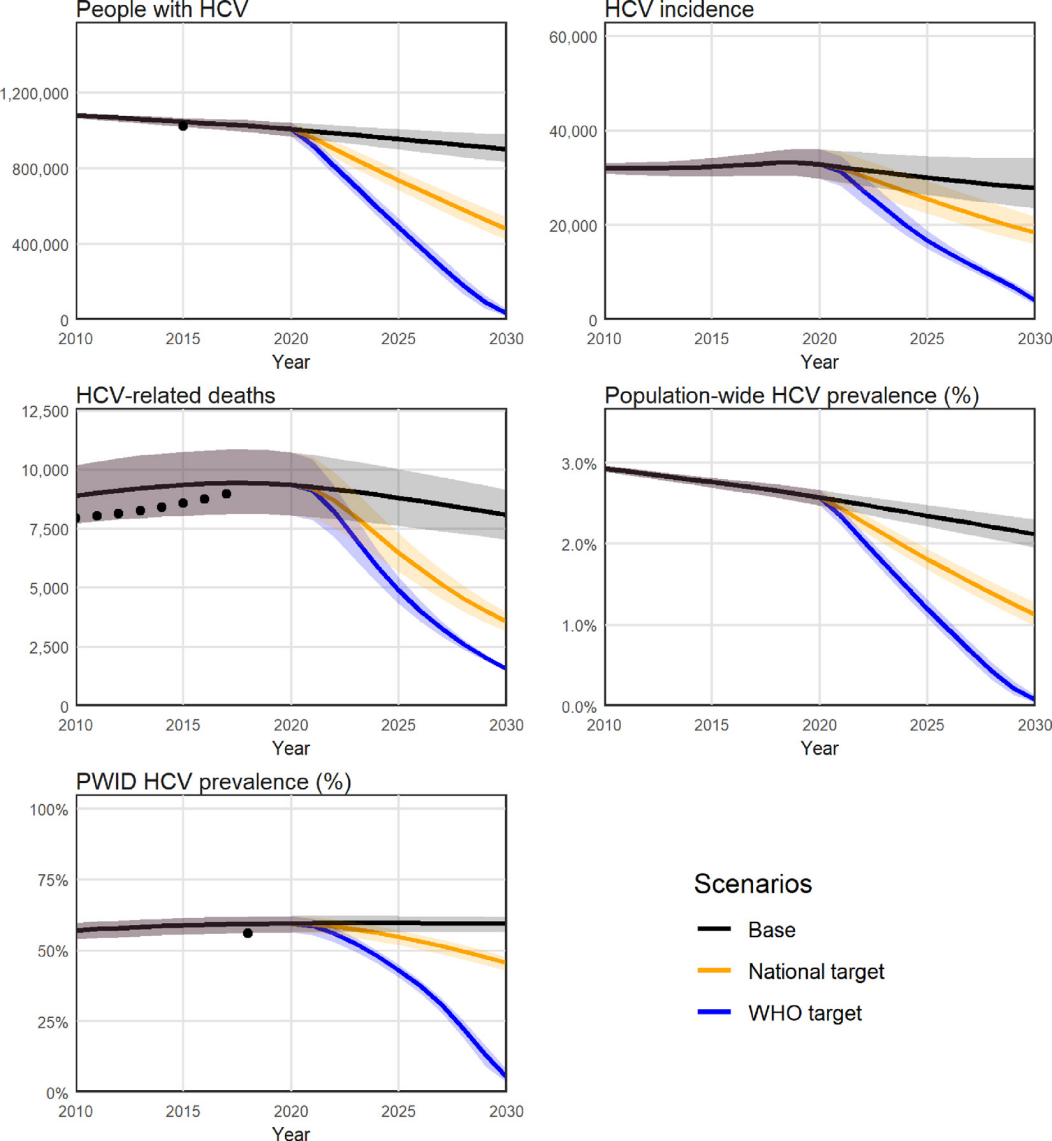
**Projected epidemiological outputs at a national level, aggregated over the 15 regional models.** Projections for (A) people living with HCV (PLHCV); (B) HCV incidence; (C) HCV-related deaths; (D) prevalence of HCV in the general population; and (E) prevalence of HCV among people who inject drugs (PWID). Black dots = data estimates; black line = baseline, orange and blue lines = testing and treatment scaled up to reach the national strategy and WHO strategy targets respectively. Uncertainty bands may decrease over time where scenarios are constrained to reach the same endpoints in 2030.

If estimated treatment numbers were maintained at approximately 4000 per year, the model projected that there would be 903,000 people with HCV in Myanmar in 2030, and between 2020 and 2030 there would be a cumulative 333,000 new HCV infections and 97,000 HCV-related deaths (Table 2 and Fig. 2). The baseline scenario was estimated to cost $100 million in testing ($11 million), treatment ($10 million) and disease management ($79 million) between 2020 and 2030, as well as $10.4 billion in lost productivity due to absenteeism (HCV-related sick days), presenteeism (people being less productive as a result of their illness) and premature deaths.

**Table 2:** summary of outcomes.

	**Status-quo**	**National strategy**	**WHO targets**
**Projections**			
People with HCV in 2030	902,700 (832,800 - 979,500)	479,700 (420,300–542,000)	33,400 (18,800–54,800)
New HCV infections 2020–2030	332,500 (292,200 - 383,800)	292,900 (260,200–332,600)	220,400 (198,300–244,300)
HCV-related deaths 2020–2030	96,500 (83,500–110,000)	72,000 (62,700–81,400)	58,100 (51,100–65,100)
New HCV infections in 2030	27,900 (23,700–34,300)	20,600 (18,100–24,000)	6500 (5100–8100)
HCV-related deaths in 2030	8100 (7000–9200)	3600 (3100–4000)	1600 (1500–1600)
Total direct costs 2020–2030 (million US$)	$100.17 ($90.76–$109.66)	$188.80 ($182.97–$194.69)	$296.72 ($282.65–$308.65)
*Testing*	$11.11 ($11.11–$11.11)	$24.79 ($24.79–$24.80)	$52.06 ($49.70–$53.91)
*Treatment*	$10.43 ($10.43–$10.43)	$115.93 ($115.93–$115.93)	$211.81 ($204.11–$217.89)
*Disease management*	$78.63 ($69.22–$88.12)	$48.08 ($42.25–$53.97)	$32.85 ($28.81–$36.88)
Indirect costs from lost productivity 2020–2030 (million US$)	$10,399 ($9081–$11,815)	$9757 ($8537–$11,066)	$9323 ($8169–$10,559)
Indirect costs from lost productivity 2020–2050 (million US$)	$26,338 ($23,080–$29,923)	$18,308 ($16,086–$20,642)	$15,362 ($13,544–$17,285)
**Epidemiological outcomes compared to status-quo**			
Deaths averted 2020–2030 relative to baseline		25% (16%–35%)	40% (33%–47%)
Mortality reduction in 2030 relative to 2015 levels		56% (51%–61%)	81% (80%–82%)
HCV infections averted 2020–2030 relative to baseline		12% (%–22%)	34% (27%–40%)
Incidence reduction in 2030 relative to 2015 levels		37% (26%–44%)	80% (75%–84%)
Reduction in people with HCV in 2030 relative to baseline in 2030		423,000 (47%) [360,600 (40%) – 482,300 (53%)]	869,200 (96%) [847,900 (94%) - 883,800 (98%)]
**Cost outcomes compared to status-quo (healthcare system perspective)**			
Additional testing / treatment / disease management costs 2020–2030 compared to baseline (million US$)		$88.64 ($82.80 - $94.52)	$196.55 ($182.48 - $208.48)
Cost per DALY averted at 2030 relative to baseline		$243 ($140–$741)	$344 ($238–$565)
**Cost outcomes compared to status-quo (societal perspective)**			
Additional testing / treatment / disease management costs 2020–2030 compared to baseline (million US$)		$88.64 ($82.80–$94.52)	$196.55 ($182.48–$208.48)
Productivity gains 2020–2030 (million US$)		$642 ($544–$748)	$1076 ($911–$1256)
Year scenario becomes cost saving		2024	2025
Net economic benefit by 2030 (million US$)		$553 ($452–$663)	$880 ($720–$1057)

### 3.2 National strategy

Reaching the national strategy targets required scaling up treatment to approximately 55,000 people each year (Appendix E). This was estimated to reduce the number of people with HCV in 2030 from 903,000 to 480,000, and over the period 2020–2030 prevent a cumulative 40,000 new infections (12%) and 25,000 HCV-related deaths (25%) compared to the baseline scenario (Table 2 and Fig. 2).

**Fig. 3:**
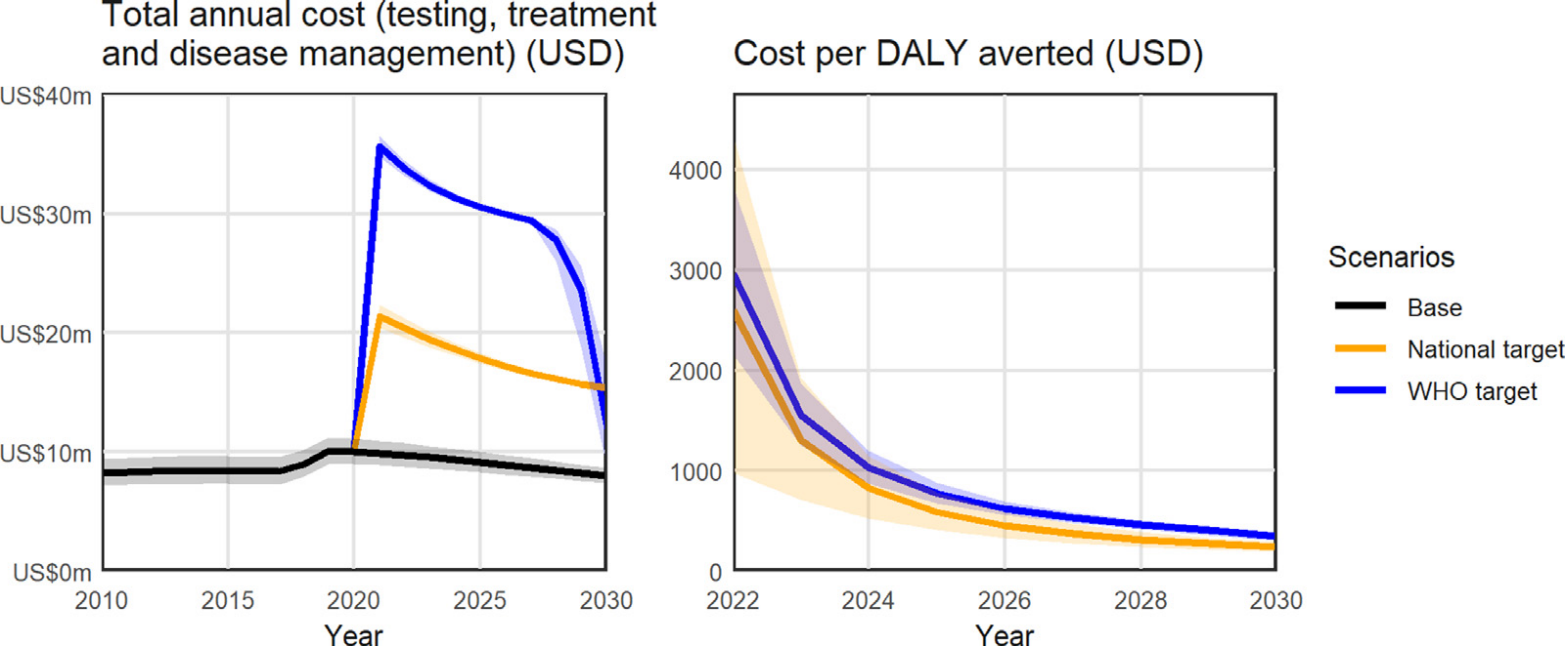
**Projected cost outputs at a national level, aggregated over the 15 regional models, from a health systems perspective**. (A) Total direct annual costs of HCV (testing, treatment and disease management) in the baseline (black), national strategy (orange) and WHO strategy (blue) scenarios; (B) Cost per disability-adjusted life year averted over time, for the national strategy (orange) and WHO strategy (blue) scenarios relative to the baseline. Uncertainty bands may decrease over time where scenarios are constrained to reach the same endpoints in 2030. Costs and DALYs are discounted at 3% per annum.

From a health systems perspective, reaching the national strategy targets was estimated to cost a total $189 million between 2020 and 2030, an additional $89 million more than the baseline ($243 per DALY averted by 2030; Fig. 3).

**Fig. 4:**
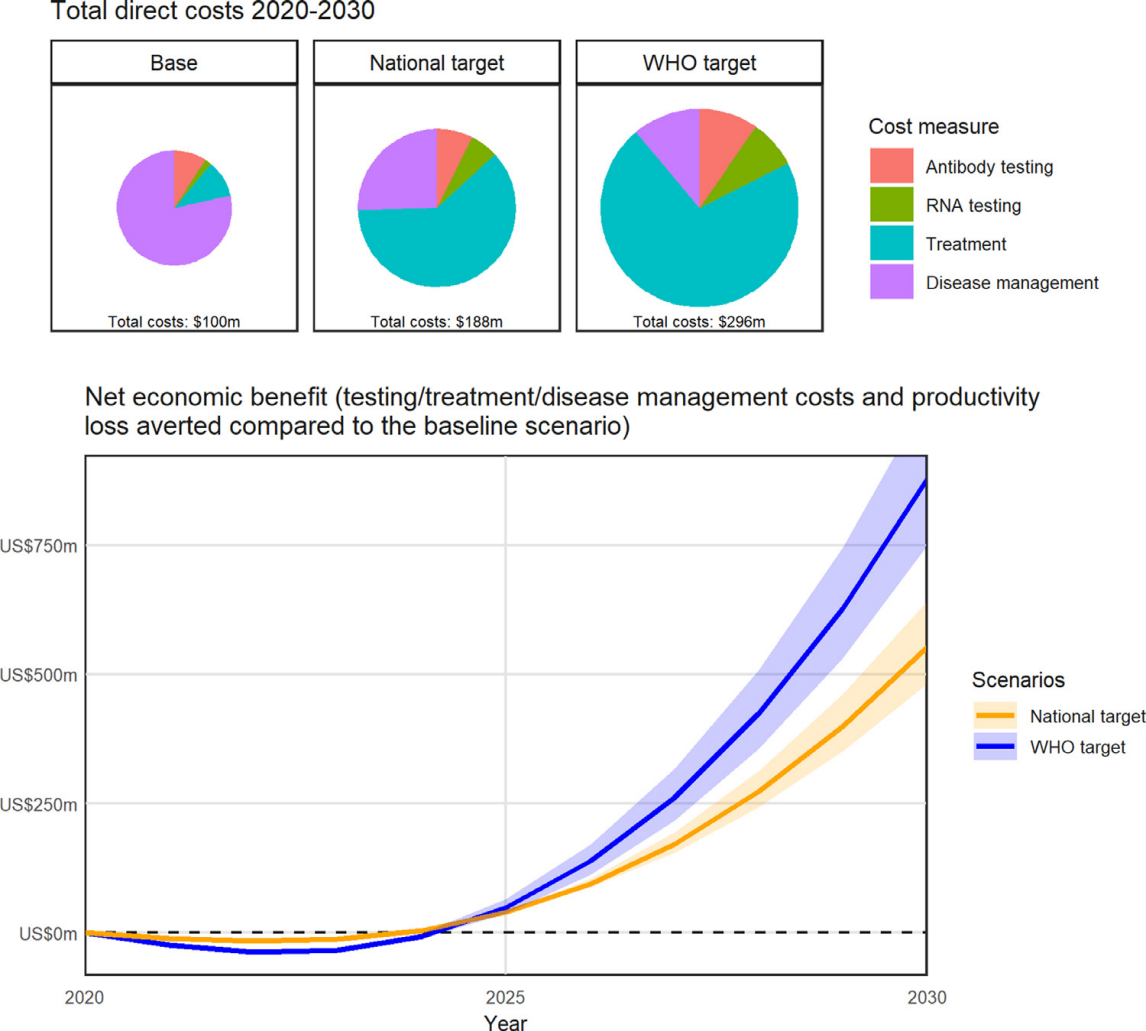
**Net economic benefit of scaling up testing/treatment to reach the national and WHO targets (at a national level, aggregated over the 15 regional models) from a societal perspective**. Difference in cumulative testing, treatment, disease management *and productivity* costs compared to the baseline.

From a societal perspective, the national strategy scenario also generated a cumulative $642 million in economic productivity gains between 2020 and 2030, making the scenario become cost-saving by 2024 with an estimated net economic benefit of $553 million by 2030 (Fig. 4).

### 3.3 WHO targets

Reaching the WHO targets required scaling up treatment to approximately 110,000 people each year (Appendix E). This was estimated to reduce the number of people with HCV in 2030 from 903,000 to 33,000, and over the period 2020–2030 prevent a cumulative 112,000 new infections (34%) and 38,000 HCV-related deaths (40%) compared to the baseline (no treatment) scenario (Table 2 and Fig. 2).

From a health systems perspective, reaching the WHO targets was estimated to cost a total $297 million between 2020 and 2030, an additional $197 million more than the baseline ($344 per DALY averted at 2030; Fig. 3).

From a societal perspective, the WHO targets scenario also generated a cumulative $1.1 billion in economic productivity gains between 2020 and 2030, making the scenario become cost-saving by 2025, with an estimated net economic benefit of $880 million by 2030 (Fig. 4).

### 3.4 Sub-national outcomes

**Fig. 5:**
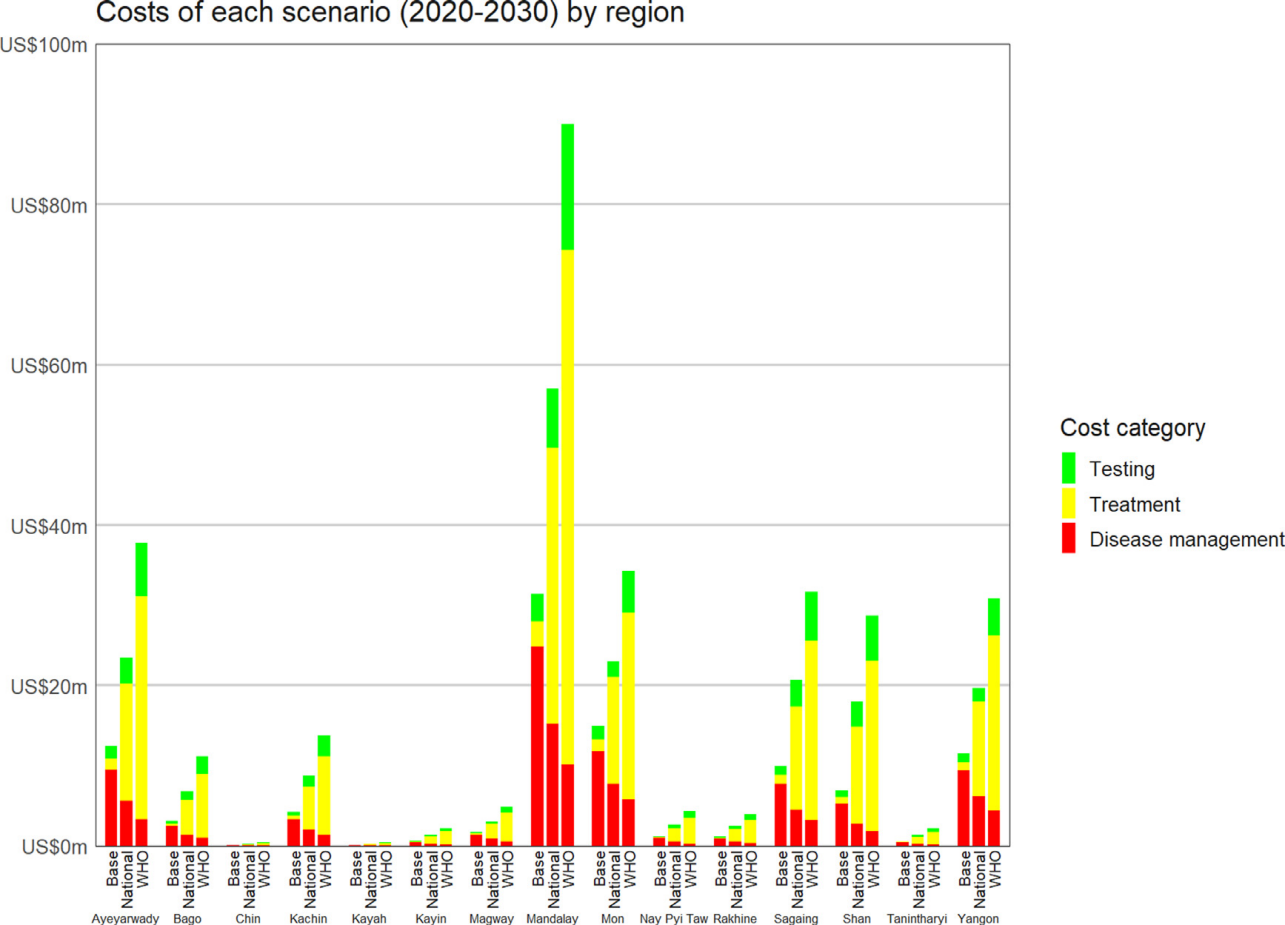
**Decomposition of the investment requirements across the 15 regional models.** Projected testing (green), treatment (yellow) and disease management (red) costs 2020–2030 for the baseline, national strategy and WHO strategy scenarios.

The largest overall HCV program (in terms of testing, treatment and disease management cost) is required for the Mandalay region, with HCV programs approximately half the size required for the Ayeyarwady region, Mon region, Sagaing region, Shan state and Yangon region (Fig. 5).

### 3.5 Sensitivity analysis

The parameters with the most significant impact on results were the cost of DAAs, the assumed test positivity rates and assumptions about the proportion of people with liver disease who accessed the health system (and incurred disease management costs) (Table 3).

**Table 3:** Outcomes of the sensitivity analysis for the National strategy.

**Scenario tested**	**Cases averted 2020–2030**	**Deaths averted 2020–2030**	**Total direct costs 2020–2030 (health system perspective) (million US$)**	**Cost per DALY averted at 2030 (health system perspective)**	**Year becomes cost-saving (societal perspective)**	**Net economic benefit at 2030 (societal perspective) (million US$)**
Point estimate	39,616	24,515	$89	$243	2024	$553
Test positivity rate is prevalence based rather than 2/prevalence	39,616	24,515	$113	$311	2025	$529
25% of people incur liver disease costs (rather that 9%)	39,616	24,515	$174	$477	2026	$468
50% of people incur liver disease costs (rather that 9%)	39,616	24,515	$308	$844	2028	$334
0% of staffing and overhead costs included compared to 50% (Ab test = $1.17; RNA test = $34.51; treatment = $86.76)	39,616	24,515	$12	$34	2023	$630
Double staffing and overhead costs included compared to 50% (Ab test = $32.59; RNA test = $34.51; treatment = $328.92)	39,616	24,515	$169	$464	2026	$473
DAAs cost $200 (compared to $86.76)	39,616	24,515	$151	$413	2025	$491
DAAs cost $40 (compared to $86.76)	39,616	24,515	$63	$173	2024	$579
Primary prevention among general population (20% reduction in probability of infection, scaled up 2020–2025)	65,952	24,548	$89	$243	2024	$561
Primary prevention among general population (40% reduction in probability of infection, scaled up 2020–2025)	88,670	24,582	$89	$242	2024	$568
Primary prevention among general population (60% reduction in probability of infection, scaled up 2020–2025)	108,564	24,612	$89	$242	2024	$574
Primary prevention PWID (20% reduction in probability of infection, scaled up 2020–2025)	50,382	24,543	$89	$243	2024	$557
Primary prevention among PWID (40% reduction in probability of infection, scaled up 2020–2025)	63,546	24,577	$89	$242	2024	$560
Primary prevention PWID (60% reduction in probability of infection, scaled up 2020–2025)	79,499	24,617	$89	$242	2024	$565

Assumptions around staffing and overhead costs had an impact on the total cost, cost per DALY averted and economic benefits, but even in a scenario with doubled staffing and overhead costs, scaling up testing and treatment to reach the national strategy targets remained cost saving from a societal perspective (by 2026) and generated a net economic benefit of $443 million by 2030 (Table 3).

When a relative reduction in incidence of 20%, 40% or 60% was assumed to be achieved through primary prevention (among the general population and among PWID), a significant number of additional infections were averted and the economic benefits increased.

## 4 Discussion

If HCV treatment continues at the current level of approximately 4000 treatments per year, we estimated that 333,000 new HCV infections and 97,000 HCV-related deaths would occur in Myanmar between 2020 and 2030, with HCV costing a total $100 million in direct costs (testing, treatment, disease management) and $10.4 billion in lost productivity due to absenteeism (HCV-related sick days), presenteeism (people being less productive as a result of their illness) and premature deaths. Scaling up testing and treatment to reach the national strategy targets of 50% of people with HCV diagnosed and 50% treated by 2030 could have a significant health impact, preventing a cumulative 40,000 new infections (12%) and 25,000 HCV-related deaths (25%). Based on testing and treatment cost data from the CT2 study, we estimate that this would cost the health system $89 million more than the status-quo, but could lead to $642 million in economic productivity gains, making the investment become cost saving from a societal perspective by 2024 with a net economic benefit of $553 million by 2030.

Reaching the WHO targets required a larger scale-up of testing and treatment than reaching the national strategy targets. While the additional cost of doing this was offset by additional longer-term benefits, significantly more upfront resources would be required. Our study suggests that the national strategy targets are ambitious in their nature and, if reached, could have significant health and economic benefits and be a major step towards elimination. This evidence supports the adoption and implementation of the National Action Plan for Viral Hepatitis Response [10] by the National Hepatitis Control Program.

It is estimated there have been about 4000 treatments a year provided in the public and non-governmental organization sectors since DAAs became available in Myanmar in 2017 [52], as well as an unknown number of treatments through the private sector (data unavailable as private clinics are not required to report treatment numbers). While these treatments are likely to have reduced morbidity and mortality for individuals who have been treated, they represent only a small fraction (7%) of what would be required to reach the national strategy targets. There are increasing examples available, including from countries in similar income brackets, that demonstrate that the substantive scale-ups required are achievable [53].

Both the national strategy and WHO target scenarios were estimated to be highly cost-effective from a health systems perspective at $243 and $344 per DALY averted respectively (17% and 24% of 2018 per capita gross domestic product, respectively [44], [54]). This is similar to other LMICs where HCV testing and treatment is highly cost-effective [11,55]. The key issue for countries such as Myanmar is the affordability of a public health response to HCV. Therefore, it is important to understand the true economic burden of HCV, as well as the benefits of elimination, to justify the prioritisation of allocating funds for HCV compared with other health issues. Our study generates this evidence. First, we followed the methods of a recent global investment framework [49] to estimate the economic burden of HCV to Myanmar from a societal perspective. This was done because considering only the health systems perspective ignores the impact of HCV on the workforce and underestimates the true economic burden. Second, we quantified the benefits of scaled up testing and treatment, finding that the additional direct costs (testing, treatment and disease management) of the national strategy and WHO target scenarios are outweighed by the gains in productivity, making both strategies cost saving from a societal perspective. This economic evidence can help to support decision-makers in the prioritisation of funds between HCV and other areas of health.

Primary prevention is an important component of HCV elimination efforts. The WHO HCV elimination targets include having, by 2030, 100% of blood donations screened, >90% of injections administered with safety-engineered devices in and out of health facilities, and >300 sterile needles and syringes provided per person who injects drugs each year [6]. Challenges in assessing baseline coverages of these interventions in LMICs, including Myanmar, make it difficult to estimate progress towards the targets, however our sensitivity analysis showed that an increased focus on primary prevention could increase the economic benefits, because as well as averting a large number of new infections they avert the productivity losses associated with them. Primary prevention could also have benefits for other diseases that are not captured in this modelling, and needle and syringe programs could also act as a point of engagement for the provision of other health services to PWID.

A strength of this analysis is that we have performed the modelling independently on the 15 states, regions and union territories of Myanmar, and aggregated the results to form national estimates. While some data inputs (e.g. HCV-related mortality) were only available at the national level and therefore applied to smaller geographical areas based on population sizes and prevalence, by modelling the individual graphical areas we have been able to control for the heterogeneities for which there is evidence, such as the estimated differences in the prevalence of HCV and injective drug use across the country. Based on the variations in prevalence estimates (among the general population and among PWID) and population sizes across the country, the results suggest that the largest overall HCV program is required for the Mandalay region, however there are additional implementation considerations that are beyond the scope of this study; for example the Kachin state has the greatest prevalence of HCV among PWID, and so more services for PWID may be needed in this area.

HIV-HCV coinfection has not been explicitly modelled due to data limitations, in particular estimates of treatment program costs and benefits. However, people with HIV-HCV coinfection are an important population group in Myanmar; among PWID there is an estimated HCV antibody prevalence of 48% and HIV-HCV coinfection prevalence of 27% [18], meaning that approximately half of all PWID with HCV also have HIV [22,26]. HCV treatment for co-infected patients (HIV-HCV) is occurring at selected HIV speciality hospitals, as well as through the USAID funded EQUIP project (with 680 patients - 89% of those completing treatment - achieving SVR12 [56]) and ongoing projects by Médecins du Monde. Importantly, HIV infection increases disease progression rates for HCV [23]. In our model we calibrated disease progression rates (and hence health gains from treatment) to represent population averages appropriate for Myanmar, however among all possible patient groups treatment programs targeted to coinfection patients could be expected to provide the greatest health benefits.

It is unclear what models of care would be used to scale-up HCV testing and treatment across Myanmar, but given the capacity constraints in tertiary hospitals (including a limited number of specialist hepatologists), it would be likely to include a mix of public and private clinics in the community. People with significant liver disease would be referred to the tertiary hospitals and current National Hepatitis Control Program (NCHP) sites, using a hub and spoke model similar to the CT2 study, with decentralization and shifting to the community setting being key to Myanmar's ability to reach its targets. The costs of testing and treatment used in this study were based on data from the CT2 study, which is similar to what is operated by other non-governmental organizations. If HCV testing and treatment were scaled up through the public health system, integration of services could reduce overhead costs compared with what we have estimated. On the other hand, capacity issues within the public health system may reduce efficiency and increase costs; for example, constrained numbers of trained healthcare workers and hepatologists and the need to share equipment may increase waiting times and lead to greater loss to follow-up. Private clinics can address some of these capacity constraints, and the role of public private partnerships in facilitating treatment scale-up (currently being piloted by the NHCP and CHAI) should be further explored. For treatments delivered through private clinics, the staffing costs may be greater than we have estimated but some of these costs would be borne by the patients. Alternatively, it could be more cost-effective to integrate HCV services into existing national tuberculosis and AIDS program testing sites, utilizing their staff and lab facilities, since these two national programs already have functioning teams in different areas and are more established than the NHCP. These factors have implications for our total cost estimates, but even in an upper bound scenario based on doubled staff and overhead costs we found that scaling up testing and treatment to reach the national strategy targets remained cost saving from a societal perspective (by 2026) and generated a net economic benefit of $443 million by 2030. It will be important to continue to evaluate the cost-effectiveness of treatment services as they are scaled up through different models of care, in order to refine these overall cost estimates.

### 4.1 Limitations

Model inputs, including epidemiological data, population data, health utilities and cost estimates come from a variety of sources, including population surveys, that and each have their own uncertainty and sampling bias. Some model inputs were only available at the national level and had to be adjusted to the regional level, and baseline treatment numbers were not available from the private sector. In addition, in some instances only single estimates for epidemiological indicators were available, meaning that there is uncertainty about underlying baseline trends.

There are some costs that have not been included in this study as they are unknown. The national strategy and WHO target scenarios do not include the costs associated with demand generation activities to increase testing, because it is currently unclear what interventions would be required to increase testing rates, and these scenarios do not include any central costs of a national implementation such as management and evaluation, data systems, program coordination and policy development. The costing model also assumes that service delivery is optimized (e.g. allows for close to 100% linkage to diagnosis and treatment, and adherence), which was the case in the CT2 site but may not extend to a broader public program, with increased loss to follow-up likely to increase testing costs and make the targets less feasible or impossible to reach. There are also some benefits that have not been included, for example the model timeframe is only up to 2030 yet liver disease progression associated with HCV is slow, meaning that testing and treatment scale-up between 2020 and 2030 will prevent cirrhosis and HCV-related mortality that would otherwise have occurred after 2030 and is hence not captured in this study.

This study used the human capital approach to estimate productivity losses associated with HCV, under the assumption that a larger workforce (e.g. from the prevention of premature deaths) would increase the economy rather than add to unemployment. Productivity losses may be underestimated as the contribution of unpaid work or the unofficial / unreported workforce to the economy were not considered. Other approaches to estimating productivity losses due to HCV, such as the friction approach, would produce lower estimates of total productivity losses because they assume that a person dying prematurely has no impact on the economy once a replacement worker is found.

## 5 Conclusion

Current levels of HCV testing and treatment in Myanmar are insufficient to reach the national strategy targets of 50% of people with HCV diagnosed and 50% treated by 2030. To reach the national targets, HCV treatment should be scaled up from an estimated 4000 per year to approximately 55,000 per year. Scaling up treatment to this level could avert 40,000 new HCV infections and 25,000 HCV-related deaths between 2020 and 2030, and from a societal perspective is likely to become cost saving by 2024 with a net economic benefit of $553 million by 2030.

## Declarations of Competing Interest

NS and the Burnet Institute received investigator-initiated research funding from Gilead Sciences for this work. KPK receives non-financial support from Mylan Myanmar, Hetero Pharmaceutical, and Royal Ruby Co. Ltd. WN received non-financial support from Mylan Myanmar and Cipla Pharmaceutical. MH receives investigator-initiated research funding from Gilead Sciences and AbbVie outside of this work. TMW, TT, HH, BD, PTZA, YX, AB, CK, SS, KSA have nothing to declare.

## Appendix Supplementary materials

Image, application 1

Image, application 2

Image, application 3

## References

[1] World Health Organization. Hepatitis C fact sheet. 2017.

[2] World Health Organization (2017). Global Hepatitis Report 2017.

[3] Stanaway J.D., Flaxman A.D., Naghavi M. (2016). The global burden of viral hepatitis from 1990 to 2013: findings from the global burden of disease study 2013. Lancet.

[4] Lawitz E., Poordad F.F., Pang P.S. (2014). Sofosbuvir and ledipasvir fixed-dose combination with and without ribavirin in treatment-naive and previously treated patients with genotype 1 hepatitis C virus infection (LONESTAR): an open-label, randomised, phase 2 trial. Lancet.

[5] Poordad F., McCone J., Bacon B.R. (2011). Boceprevir for untreated chronic HCV genotype 1 infection. N. Engl. J. Med..

[6] World Health Organization. Global health sector strategy on viral hepatitis 2016-2021. Accessed 22 January 2021 from: http://www.who.int/hepatitis/strategy2016-2021/ghss-hep/en/.

[7] McHugh M., Wu A., Chevaliez S., Pawlotsky J., Hallin M., Templeton K. (2017). Multicenter evaluation of the cepheid xpert hepatitis C virus viral load assay. J. Clin. Microbiol..

[8] Gupta E., Agarwala P., Kumar G., Maiwall R., Sarin S.K. (2017). Point-of-care testing (POCT) in molecular diagnostics: performance evaluation of GeneXpert HCV RNA test in diagnosing and monitoring of HCV infection. J. Clin. Virol..

[9] Lwin Aye Aye, A K.S., Htun Moh Moh, Kyaw Yi Yi, Zaw Ko Ko, Aung Toe Thiri, Kyaw Myat Phone, Kyi Khin Pyone, Thant Kyaw Zin (2017). Sero-prevalence of hepatitis B and C viral infections in Myanmar: national and regional survey in 2015. Myanmar Health Sci. Res. J..

[10] National Hepatitis Control Program, Department of Public Health. Myanmar national action plan for viral hepatitis response 2017–2020, 2017.

[11] Scott N., Mohamed Z., Rwegasha J., Mbwambo J., Lemoine M., Hellard M. (2019). Upscaling prevention, testing and treatment to control hepatitis C as a public health threat in Dar es Salaam, Tanzania: a cost-effectiveness model. Int. J. Drug Policy.

[12] Pozzetto B., Memmi M., Garraud O., Roblin X., Berthelot P. (2014). Health care-associated hepatitis C virus infection. World J. Gastroenterol..

[13] Ver Hoeve E., Codlin A., Jawed F. (2013). Persisting role of healthcare settings in hepatitis C transmission in Pakistan: cause for concern. Epidemiol. Infect..

[14] Draper B.L., Pedrana A., Howell J. (2020). Decentralized, community-based hepatitis C point-of-care testing and direct-acting antiviral treatment for people who inject drugs and the general population in myanmar: protocol for a feasibility study. JMIR Res. Protoc..

[15] Draper B., Yee W.L., Pedrana A. (2020). Community-based point-of-care hepatitis C testing and general practitioner initiated direct-acting antiviral therapy in Yangon, Myanmar (CT2 study). J. Hepatol..

[16] Burnet Institute. Eliminating hepatitis C in Myanmar. Accessed 22 January 2021 from https://www.burnet.edu.au/system/asset/file/4418/Eliminating_HepC_in_Myanmar.pdf. 2020.

[17] Myanmar National AIDS Program. Integrated biological and behavioural surveillance survey and population size estimates among people who inject drugs 2014.

[18] National AIDS Program, Ministry of Health and Sports Myanmar. Myanmar integrated biological and behavioural surveillance survey & population size estimates among people who inject drugs (PWID) 2017–2018. Accessed 15 April 2019 from: https://www.aidsdatahub.org/sites/default/files/highlight-reference/document/Myanmar_IBBS_and_Population_size_estimates_among_PWID_2017-2018.pdf. 2019.

[19] Myanmar Ministry of Health. Myanmar national strategic plan on HIV and AIDS 2011–2015. 2011.

[20] World Health Organization. ATLAS of substance use disorders. Resources for the prevention and treatment of substance use disorders (SUD). 2010.

[21] Nelson P.K., Mathers B.M., Cowie B. (2011). Global epidemiology of hepatitis B and hepatitis C in people who inject drugs: results of systematic reviews. Lancet N. Am. Ed..

[22] Zhou Y.-.H., Liu F.-.L., Yao Z.-.H. (2011). Comparison of HIV-, HBV-, HCV-and co-infection prevalence between Chinese and Burmese intravenous drug users of the China-Myanmar border region. PLoS One.

[23] Graham C., Baden L., Yu E. (2001). Influence of human immunodeficiency virus infection on the course of hepatitis C virus infection: a meta-analysis. Clin. Infect. Dis..

[24] Scott N., McBryde E., Thompson A., Doyle J., Hellard M. (2017). Treatment scale-up to achieve global HCV incidence and mortality elimination targets: a cost-effectiveness model. Gut.

[25] Scott N., Doyle J., Wilson D.P. (2017). Reaching hepatitis C virus elimination targets requires health system interventions to enhance the care cascade. Int. J. Drug Policy.

[26] Aye N., Oo M., Harries A. (2018). HIV, HBV and HCV in people who inject drugs and are placed on methadone maintenance therapy, Yangon, Myanmar. Public Health Action.

[27] Micallef J.M., Kaldor J.M., Dore G.J. (2006). Spontaneous viral clearance following acute hepatitis C infection: a systematic review of longitudinal studies. J. Viral Hepat..

[28] Mondelli M.U., Cerino A., Cividini A. (2005). Acute hepatitis C: diagnosis and management. J. Hepatol..

[29] Gane E.J., Stedman C.A., Hyland R.H. (2014). Efficacy of nucleotide polymerase inhibitor sofosbuvir plus the NS5A inhibitor ledipasvir or the NS5B non-nucleoside inhibitor GS-9669 against HCV genotype 1 infection. Gastroenterology.

[30] Gane E.J., Stedman C.A., Hyland R.H. (2011). Once daily PSI-7977 plus RBV: pegylated interferon-alfa not required for complete rapid viral response in treatment-naive patients with HCV GT2 or GT3. Hepatology.

[31] Poordad F., Lawitz E., Kowdley K.V. (2013). Exploratory study of oral combination antiviral therapy for hepatitis C. N. Engl. J. Med..

[32] Thein H.H., Yi Q., Dore G.J., Krahn M.D. (2008). Estimation of stage-specific fibrosis progression rates in chronic hepatitis C virus infection: a meta-analysis and meta-regression. Hepatology.

[33] National Centre in HIV Epidemiology and Clinical Research (2010). Epidemiological and Economic Impact of Potential Increased Hepatitis C Treatment Uptake in Australia.

[34] Nahon P., Bourcier V., Layese R. (2017). Eradication of hepatitis C virus infection in patients with cirrhosis reduces risk of liver and non-liver complications. Gastroenterology.

[35] Martin N.K., Devine A., Eaton J.W. (2014). Modeling the impact of early antiretroviral therapy for adults coinfected with HIV and hepatitis B or C in South Africa. AIDS.

[36] Salomon J.A., Vos T., Hogan D.R. (2012). Common values in assessing health outcomes from disease and injury: disability weights measurement study for the global burden of disease study 2010. Lancet N. Am. Ed..

[37] Shepherd J., Jones J., Hartwell D., Davidson P., Price A., Waugh N. (2007). Interferon alfa (pegylated and non-pegylated) and ribavirin for the treatment of mild chronic hepatitis C: a systematic review and economic evaluation. Health Technol. Assess. (Rockv).

[38] Salomon J.A., Haagsma J.A., Davis A. (2015). Disability weights for the global burden of disease 2013 study. Lancet Glob. Health.

[39] WHO Global Health Observatory. Life tables by country; Myanmar. Accessed 17 Feb 2021 from: https://apps.who.int/gho/data/view.main.61130. 2019.

[40] Mathers B.M., Degenhardt L., Bucello C., Lemon J., Wiessing L., Hickman M. (2013). Mortality among people who inject drugs: a systematic review and meta-analysis. Bull. World Health Organ..

[41] Myanmar Information Management Unit. The 2014 Myanmar population and housing census, 2016.

[42] IHME Global Burden of Disease Estimates. Accessed 23 July 2019 from: http://ghdx.healthdata.org/gbd-2019.

[43] Chhatwal J., Chen Q., Bethea E.D. (2018). Hep C calculator: an online tool for cost-effectiveness analysis of DAAs. Lancet Gastroenterol. Hepatol..

[44] The World Bank. GDP per capita estimates. Accessed 23 July 2019 from: https://data.worldbank.org/indicator/NY.GDP.PCAP.CD?name_desc=false. 2018.

[45] The World Bank. Employment to population ratio. Available from: https://data.worldbank.org/indicator/SL.EMP.TOTL.SP.ZS. 2016.

[46] Dibonaventura M.D., Wagner J.-.S., Yuan Y., L'Italien G., Langley P., Ray Kim W. (2011). The impact of hepatitis C on labor force participation, absenteeism, presenteeism and non-work activities. J. Med. Econ..

[47] Younossi Z., Brown A., Buti M. (2016). Impact of eradicating hepatitis C virus on the work productivity of chronic hepatitis C (CH-C) patients: an economic model from five European countries. J. Viral Hepat..

[48] World Health Organization. Disease burden and mortality estimates; cause-specific mortality, 2000–2016. Available from: http://www.who.int/healthinfo/global_burden_disease/estimates/en/. 2016.

[49] Scott N., Kuscel C., Pedrana A. (2020). A model of the economic benefits of global hepatitis C elimination: an investment case. Lancet Gastroenterol. Hepatol..

[50] Personal communication with specialists providing care to patients with hepatitis C in Myanmar. 2020.

[51] Grossman M. (1972). On the concept of health capital and the demand for health. J. Polit. Econ..

[52] Personal communication with the Myanmar national hepatitis control programme 2020.

[53] Schröeder S.E., Pedrana A., Scott N. (2019). Innovative strategies for the elimination of viral hepatitis at a national level: a country case series. Liver Int..

[54] The World Bank. GDP per capita. Accessed 1 May 2018 from: https://data.worldbank.org/indicator/NY.GDP.PCAP.CD?name_desc=false. 2018.

[55] Chhatwal J., He T., Lopez-Olivo M.A. (2016). Systematic review of modelling approaches for the cost effectiveness of hepatitis C treatment with direct-acting antivirals. Pharmacoeconomics.

[56] Barralon M., Cavenaugh C., Chasela C., et al. Equip policy report: treatment outcomes and costs of a simplified antiviral treatment strategy for hepatitis C in Myanmar. Accessed 27 May 2020 from: https://sites.bu.edu/hiv/files/2019/12/Myanmar-EQUIP-Myanmar-HCV_FinaL.pdf. 2019.

