# Supplementary material for: Hepatitis C elimination in Myanmar: Modelling the impact, cost, cost-effectiveness and economic benefits
Source: Lancet Reg Health West Pac. 2021 Mar 23;10:100129. doi: 10.1016/j.lanwpc.2021.100129 (PMC8315611; doi:10.1016/j.lanwpc.2021.100129)
Supplement: Supplementary file 1 [file mmc1.pdf]

Supplementary material

Hepatitis C elimination in Myanmar: modelling the impact, cost, cost-effectiveness and economic benefits

Appendix A: model description

We used an open deterministic differential equation model of HCV transmission, liver disease progression and the cascade of care based on Scott et al. [1,2] (Figure 1), which was set up for each of the 15 geographical divisions (states, regions and union territories) of Myanmar. The model was implemented in Python v3.8.

Populations
The model considers four population groups: general population 15-64 years, general population 65+ years, PWID, former PWID. PWIDs could become former PWIDs due to cessation of injecting, and people from the general population can be recruited to become PWID.

Infection status
Individuals were classified as either susceptible (infection naïve or previously achieving spontaneous clearance or sustained viral response through treatment), acutely infected or chronically infected.

Transmission
Transmission was modelled to occur among PWID as well as among the rest of the general population. Susceptible PWID became infected at a rate proportional to the time-varying HCV prevalence among PWID, and susceptible people in the general population becoming infected at a rate proportional to the time-varying HCV prevalence among the general population in the model. Following infection, people were modelled to experience a short duration of acute infection, after which a proportion spontaneously cleared the virus and became susceptible again without treatment, and the remaining proportion became chronically infected.

Liver disease
METAVIR scores were used to classify everyone in the model according to stages of liver disease: F0, F1, F2, F3, F4, decompensated cirrhosis (DC), hepatocellular carcinoma (HCC) or post liver transplant. Liver disease progression was only modelled to occur for chronically infected individuals, with the exception of susceptible individuals with compensated cirrhosis (F4) who could develop DC or HCC.

Care cascade
Individuals who were chronically infected were also classified according to their care cascade status: infected and undiagnosed, infected and diagnosed antibody positive, infected and diagnosed RNA-positive, on treatment, or failed treatment.

Testing and treatment
In order for infected people to become cured, they need to be antibody tested, RNA tested and treated. Following cure, people in the model retained their liver disease (i.e. regression of disease was not included), and reinfection was allowed to occur among PWID at the same rate as initial infection (although re-infected PWID required an RNA test for screening). The number of tests and treatment available in each scenario and user inputs for the projection period.

Mortality
All-cause mortality was modelled to occur for everyone in the model, and additional injecting-related mortality was included for PWID and additional liver-related mortality was included for people with DC or HCC.

Calibration
For each region, the model was calibrated to time series data on the prevalence of HCV among the general population, the prevalence of HCV among PWID, the annual number of HCV-related deaths, the total number of people living with HCV, the estimated incidence of HCV, and the proportion of people living with HCV who were diagnosed. This involved simultaneously varying parameters for: the force of infection among PWID (the force of infection was dynamic and dependent on prevalence, but a constant scalar factor was varied), the average length of injecting career among PWID, the disease progression rates (F0→F1, F1→F2, F3→F4, F4→DC, F4→HCC, DC→HCC), the annual probability of dying from DC, the annual probability of dying from HCC, and the annual probability of having an HCV antibody test for each population group.

Productivity gains from people cured of HCV
The epidemiological model described above was used to capture direct costs (testing, treatment and disease management), but to estimate economic productivity losses an additional calculations were required. This module took as inputs the annual numbers people with HCV and people who had been cured from HCV, and produced the annual productivity losses from absenteeism and presenteeism (Figure S1).

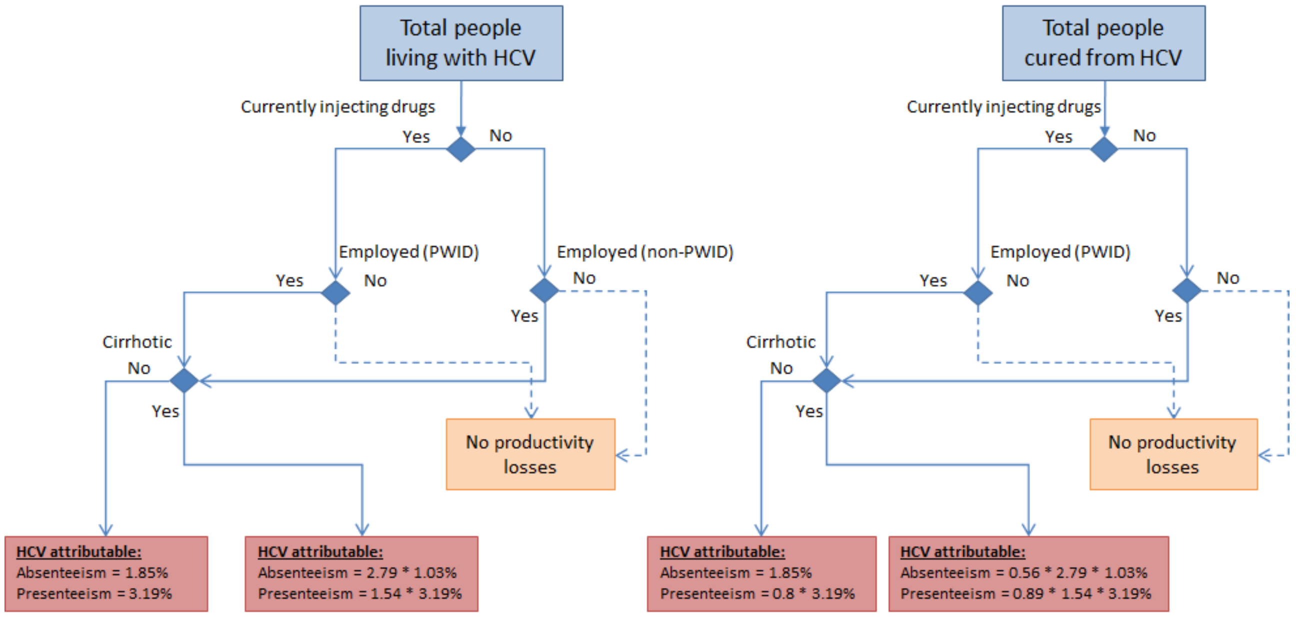

Figure S1: Schematic of productivity model.

Productivity gains from averted deaths
Productivity gains from deaths averted were included. However, a disproportionate amount of HCV-related deaths are estimated to occur among older age groups, and therefore only a fraction of these averted deaths were assumed to result in years of productive life gained. For each year in the projection timeframe, the productive life gained from deaths averted in that year were calculated by assuming:

- The fraction of averted deaths among the 60+ age category did not produce additional years of productivity
- Of the fraction of averted deaths among the 50-59 age category:
    - All of them contributed an additional year of productivity in the year they occurred;

    - 8/9th of these deaths contributed an additional year of productivity the year after they occurred (approximating 1/9th of this age band entering non-productive life at 60 years)
    - 7/9th of these deaths contributed an additional year of productivity two years after they occurred;
    - And so on, with the fraction of deaths averted from this age category contributing decreasing productivity gains for the next 9 years, before no longer producing additional productive years.
- Of the fraction of averted deaths among the 30-49 age category, the methodology above was used to attribute their ongoing productive years following the year that their death was prevented.

Years of productive life lost due to premature death were converted to economic outcomes using per capita gross domestic product, scaled by the employment rate.

Future economic productivity gains were discounted at 3%.

Appendix B: Summary of data inputs for the 15 states, regions and union territories of Myanmar

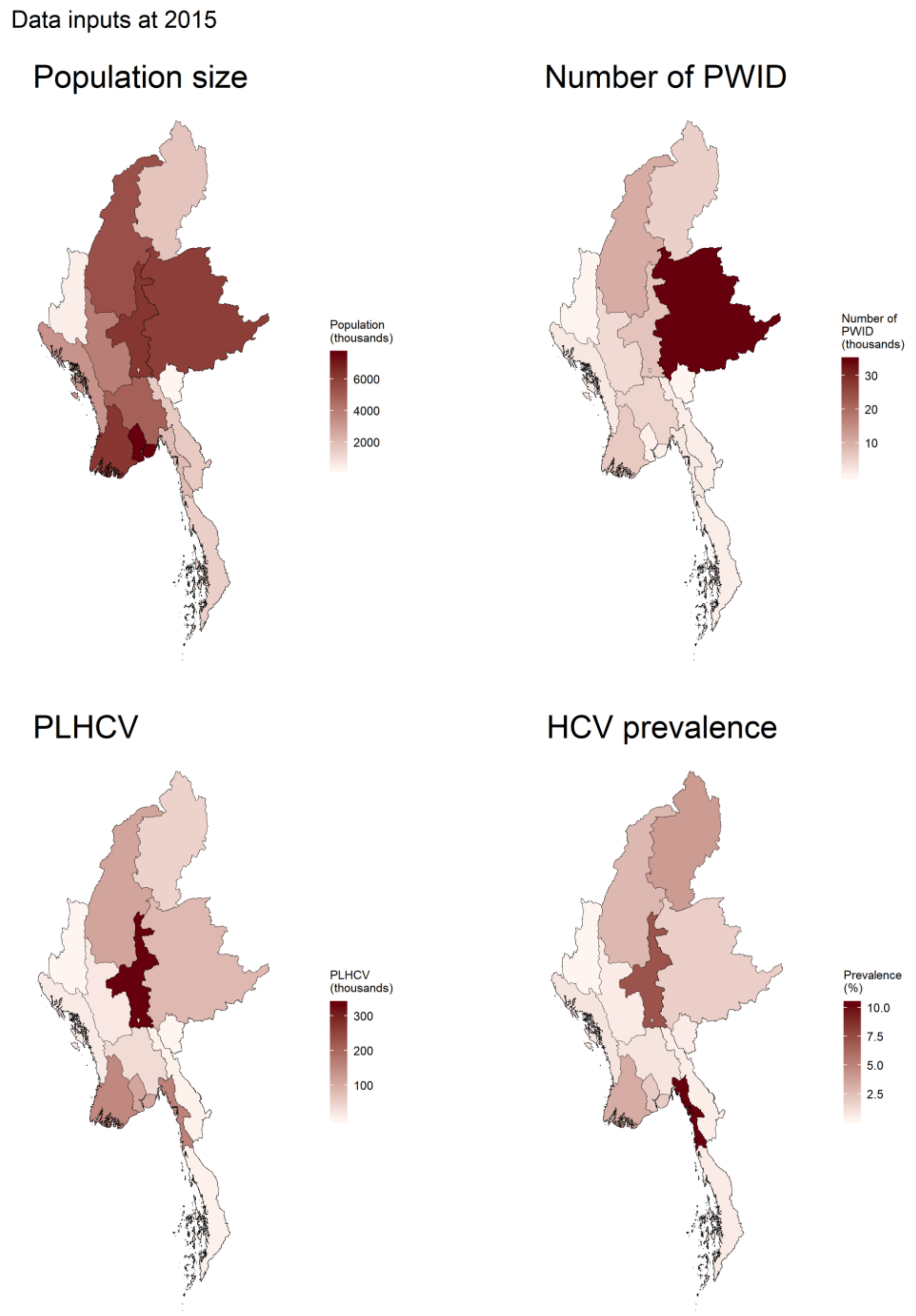

Figure S2: Data inputs for the sub-national hepatitis C models. PLHCV = people living with hepatitis C; PWID = people who inject drugs.

Table S1: Data inputs for the sub-national hepatitis C models. Description of calculations used to generate inputs, as well as the median, interquartile range and range of data inputs across regions.

Region-specific characteristics		
15-64 year old population size	Time varying. At start of 2014: Median 1,305,561 across regions (range 176,934-	Myanmar Information Management Unit [3]

	5,219,941; IQR 858,419-3,712,196)	
PWID population size	Time varying. At start of 2018: Median 4,239 across regions (range 388- 31,853; IQR 1,726-6,785)	Limited data on PWID population size estimates in different states [4].<br>For the five states with data in their cities available, proportion of PWID in cities were used to represent the proportion of PWID in the state.<br>For the rest of the states, anti-HCV prevalence was assumed to be the same in each state and calculated the population weighted average proportion of PWID in the cities with estimated number of PWID available.<br>The final proportion of PWID in each state was scaled down by the total number of PWID calculated via above method over the national estimate of number of PWID, i.e. anti-HCV prevalence in each state= Raw estimated proportion of PWID in the state/ (national PWID number/ raw estimated total number of PWID).
Total people living with hepatitis C (PLHCV)	Time varying. At start of 2014: Median 34,857 across regions (range 1,147- 326,964; IQR 6,872- 110,145)	The sero-prevalence of anti-HCV was measured in a national and regional sero-survey in 2015 [5]. It was estimated the sero-prevalence in 2014 were the same as what's in 2015.<br>Total people living with hepatitis C in each state in 2014= anti-hepatitis C prevalence in 2015* population in each state in 2014 * (1-0.26).<br>Population size were sourced from Myanmar census data [3]
Hepatitis C-related mortality	Time varying. At start of 2016: 8760	HCV-related liver cancer deaths was sourced from 2017 Global Burden of Disease study [6]. Total national deaths were distributed across regions based on the estimated PLHCV.
HCV Ab+ prevalence among PWID	Time varying. At start of 2018: Median 39% across regions (range 27%- 84%)	For 2018, the Integrated Biological and Behavioural Surveillance Survey among PWID [4] reported anti-HCV prevalence among PWID in thirteen cities/towns located in five administrative regions. Estimates were formed for the 5 regions by pooling multiple cities/towns where applicable. A national estimate was made based on population-weighted averages from each of the 5 regions, which was applied to the regions with missing data.
HCV Ab+ prevalence in general population	Time varying. At start of 2015: Median 1.3% across regions (range 0.3%- 10.3%; IQR 0.7%-3.3%)	For 2015, National and regional survey [5].
Incidence	Time varying. At start of 2016: Median 1,628 across regions (range 54- 15,275; IQR 321-5,146)	Global Burden of Disease 2017 [6]. State-specific estimates were distributed across regions based on the estimated PLHCV.

Appendix C: Additional details of cost estimation

Table S2: Calculations of unit costs for items in the CT2 study

Item	Unit cost (2018 US$)	Calculations / notes
Lab related unit costs		
Antibody testing		
HCV antibody test	$1.17	Unit cost = (10 mins lab staff time per appointment) * ($4620 annual lab staff salary)/(48 weeks per year * 5 days per week * 7 hours per day * 60 minutes per hour)) + HCV Ab test costs = $0.46 + $0.71
RNA testing		
HCV RNA test	$34.51	Lab staff costs (as per antibody test) + test cost + overhead costs = $0.46 + $22.00 + $12.05. Overhead costs include fractional costs for purchase of a Cepheid Xpert machine, centrifuge and disposal costs. For each "overhead" items in our analysis, if they were annual costs then the total cost was divided by the estimated number of appointments per year to determine a per-appointment fractional cost. For once off costs, the total cost was divided by the estimated number of appointments and the estimated number of years that the item/machine can last (in this analysis we depreciated the Xpert machine over 2-years).
Treatment eligibility		
**Total CT2 lab package test**	**$36.53**	
HIV RDT	$1.83	Lab staff costs (as per antibody test) + test cost = $0.46 + $1.37.
HBV sAg RDT	$1.10	Lab staff costs (as per antibody test) + test cost = $0.46 + $0.64.
Other lab tests including LFT	$32.98	Lab staff costs + lab items (consumables such as Dettol, cotton, needle, syringe, EDTA tubes, etc) + transport + test costs = $0.46 + $0.39 + $2.13 + $30.00
Pregnancy test	$0.63	Lab staff costs (as per antibody test) + test cost = $0.46 + $0.17.
SVR 12		
HCV RNA test (SVR 12)	$34.46	Lab staff costs (as per antibody test, but only 9 minutes) + test cost + overheads = $0.41 + $22.00 + $12.05
Clinic set up costs		
Total office set up costs attributable to each appointment	***$9.04***	
Internet	$0.29	Monthly cost multiplied by 12 (annual cost = $351.96) and divided by appointments per year
Electricity bill	$0.73	Monthly cost multiplied by 12 (annual cost = $879.96) and divided by appointments per year
Phone Bill	$0.07	Monthly cost multiplied by 12 (annual cost = $79.92) and divided by appointments per year
Generator	$0.27	Once off cost ($986.66) divided by appointments per year and warranty years
Refrigerator	$0.12	Once off cost ($565.00) divided by appointments per year and warranty years

IT infrastructure cost	$2.24	Once off cost ($4034.81) divided by appointments per year and warranty years
Room Rent	$5.32	Monthly cost multiplied by 12 (annual cost = $6399.96) and divided by appointments per year
Staff costs		
Salary		
Lab staff annual salary	$4620 (annual cost)	Annual lab staff salary has been included in the calculation of unit cost for lab tests
Nurse annual salary	$7236 (annual cost)	Unit costs for different types of visits are calculated as:<br>(average time per visit in hours) * (staff annual salary) / (staff annual working hours [7 hours per day, 5 days per week, 48 weeks per year])
Doctor annual salary	$9240 (annual cost)	Unit costs for different types of visits are calculated as:<br>(average time per visit in hours) * (staff annual salary) / (staff annual working hours [7 hours per day, 5 days per week, 48 weeks per year])
Administrative staff annual salary	$2760 (annual cost)	Unit costs for different types of visits are calculated as:<br>(average time per visit in hours) * (staff annual salary) / (staff annual working hours [7 hours per day, 5 days per week, 48 weeks per year])
Salary costs attributable to each appointment		
Initial consult	$5.27	Nurse (40 mins) + Doctor (25 mins) + administrative staff (4 mins)<br>= $2.87 + $2.29 + $0.11
Follow-up consult	$0.97	Nurse (3 mins) + Doctor (7 mins) + administrative staff (4 mins)<br>= $0.22 + $0.64 + $0.11
SVR12 consult	$1.30	Nurse (13 mins) + Doctor (4 mins)<br>= $0.93 + $0.37
Training		
Health care worker training attributable to each appointment	$2.29	Unit cost = total training cost ($2748) / lifetime (each staff has one training, with an average staff length of employment of 1 year) * (appointments they have per year)<br>Lab staff in CT2 study actually attended three trainings, but two were funded by a donor.

Table S3: Calculations of unit costs for testing and treatment interactions in the model

Interaction	Average cost	Breakdown of cost
HCV Ab test	**$16.88**<br>= $1.17 + $5.27 + $2.29 + $9.04	Unit cost for HCV antibody test + staff time cost (initial consult) + staff training cost + office set up cost
HCV RNA test	**$34.51**	Unit cost for HCV RNA test. Reflexive testing does not incur additional staff costs (except lab staff, which is included in test).
12-week treatment course	**$207.28**<br>= $36.53 + $0.97*3 + ($34.46 + $1.30) + $2.29*4 + $9.04*4 + $86.76	Unit cost for CT2 lab package test (treatment eligibility) + unit costs for follow-up visits (3 times) + unit cost for SVR12 visit + staff training cost*4 times (3 follow up visits plus SVR 12 visit) + office set up cost*4 times + drug cost for 12-week regime

Note: costs of hepatologist and HBV screening have not been included as part of this model of care as they were required for so few patients.

Appendix D: Sub-national model projections

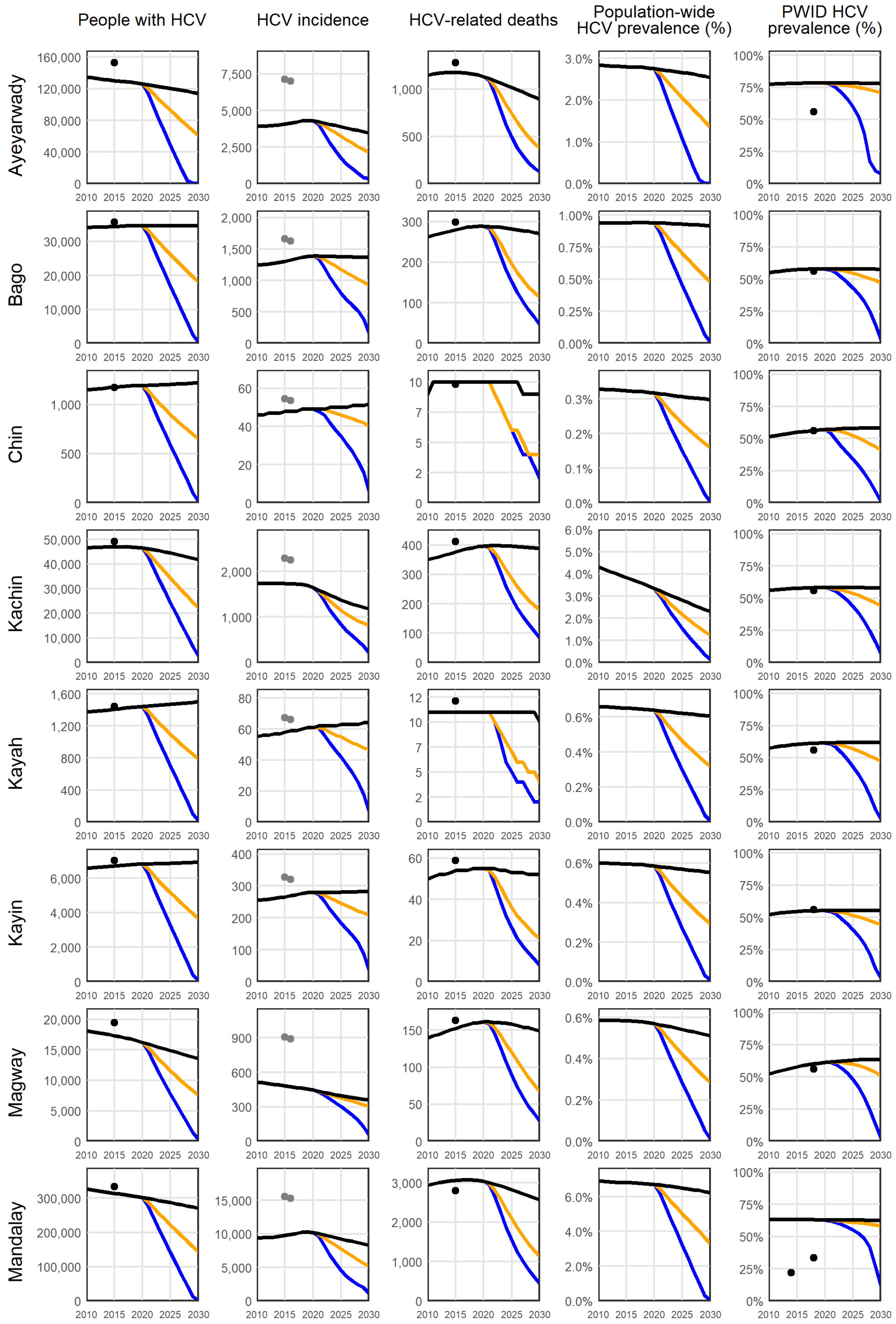

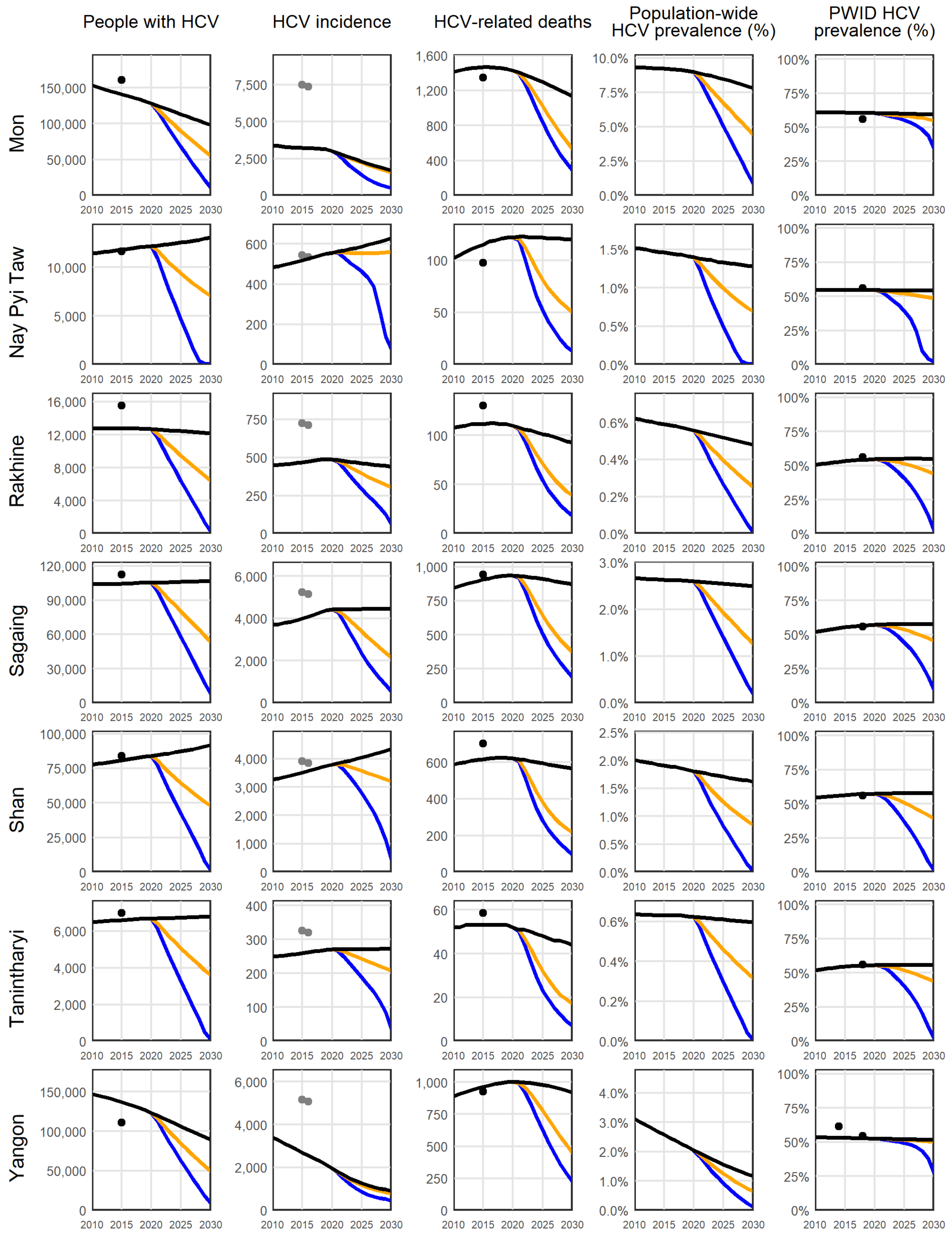

Figure S3: Projected epidemiological outputs for the 15 regional models. Projections for (column 1) people living with HCV (PLHCV); (column 2) HCV incidence; (column 3) HCV-related deaths; (column 4) prevalence of HCV in the general population; and (column 5) prevalence of HCV among people who inject drugs. Black line = baseline, orange and blue lines = testing and treatment scaled up to reach the national strategy and WHO strategy targets respectively.

Table S4: Model outputs for each scenario, by region.

	Baseline	National strategy	WHO targets		Baseline	National strategy	WHO targets
Ab tests				Total direct costs 2020-2030 (million US$)			
Ayeyarwady	34,232	54,792	107,357	Ayeyarwady	$12.47	$23.48	$37.76
Bago	7,975	16,835	34,292	Bago	$3.14	$6.81	$11.15
Chin	264	594	1,281	Chin	$0.11	$0.25	$0.42
Kachin	11,022	22,912	43,638	Kachin	$4.28	$8.75	$13.79
Kayah	319	759	1,468	Kayah	$0.12	$0.29	$0.48
Kayin	1,573	3,883	6,966	Kayin	$0.60	$1.38	$2.23
Magway	4,367	4,817	12,414	Magway	$1.75	$3.04	$4.91
Mandalay	74,833	123,283	258,954	Mandalay	$31.40	$56.98	$89.98
Mon	35,992	29,812	87,602	Mon	$14.94	$23.01	$34.25
Nay Pyi Taw	2,607	7,257	13,623	Nay Pyi Taw	$1.19	$2.70	$4.32
Rakhine	3,487	6,817	12,211	Rakhine	$1.20	$2.48	$4.00
Sagaing	25,212	56,772	99,832	Sagaing	$9.95	$20.71	$31.67
Shan	18,788	48,418	88,845	Shan	$6.91	$17.96	$28.76
Tanintharyi	1,573	3,403	6,681	Tanintharyi	$0.58	$1.33	$2.18
Yangon	24,849	27,979	76,909	Yangon	$11.55	$19.65	$30.82
RNA tests				Productivity losses (million US$)			
Ayeyarwady	7,370	44,080	99,264	Ayeyarwady	$1,267.15	$1,191.41	$1,124.95
Bago	1,716	15,253	31,784	Bago	$324.49	$300.74	$287.11
Chin	55	605	1,324	Chin	$11.86	$10.96	$10.77
Kachin	2,376	18,456	37,098	Kachin	$446.22	$415.30	$396.47
Kayah	66	726	1,405	Kayah	$13.44	$12.44	$11.85
Kayin	341	3,171	6,305	Kayin	$62.63	$57.91	$54.92
Magway	935	5,016	12,061	Magway	$177.04	$166.24	$157.93
Mandalay	16,115	101,795	226,218	Mandalay	$3,361.59	$3,160.01	$3,012.45
Mon	7,755	29,492	69,715	Mon	$1,557.85	$1,481.40	$1,433.10
Nay Pyi Taw	561	6,756	13,203	Nay Pyi Taw	$131.82	$120.81	$112.16
Rakhine	748	5,268	11,063	Rakhine	$124.12	$115.80	$111.09
Sagaing	5,434	44,224	84,805	Sagaing	$1,040.51	$965.89	$926.76
Shan	4,048	47,229	86,180	Shan	$748.32	$687.29	$655.65
Tanintharyi	341	3,131	6,226	Tanintharyi	$59.75	$55.42	$52.89
Yangon	5,357	23,237	64,209	Yangon	$1,072.43	$1,015.50	$974.98
Treatment				Return on investment by 2030 (million US$)			
Ayeyarwady	6,952	70,172	133,740	Ayeyarwady		$64.74	$116.91
Bago	1,617	20,957	38,475	Bago		$20.08	$29.36
Chin	55	785	1,404	Chin		$0.76	$0.78
Kachin	2,233	25,883	46,985	Kachin		$26.46	$40.24
Kayah	66	936	1,699	Kayah		$0.82	$1.23
Kayin	319	4,259	7,828	Kayin		$3.94	$6.08
Magway	891	8,871	16,969	Magway		$9.52	$15.95
Mandalay	15,202	165,222	308,858	Mandalay		$176.00	$290.56
Mon	7,315	63,955	111,869	Mon		$68.37	$105.44
Nay Pyi Taw	528	8,168	15,265	Nay Pyi Taw		$9.49	$16.52
Rakhine	704	7,534	13,856	Rakhine		$7.04	$10.23
Sagaing	5,115	61,815	107,656	Sagaing		$63.86	$92.03
Shan	3,817	58,157	102,231	Shan		$49.98	$70.81
Tanintharyi	319	4,209	7,714	Tanintharyi		$3.58	$5.25
Yangon	5,049	56,839	104,572	Yangon		$48.82	$78.17

Appendix E: Additional model outputs

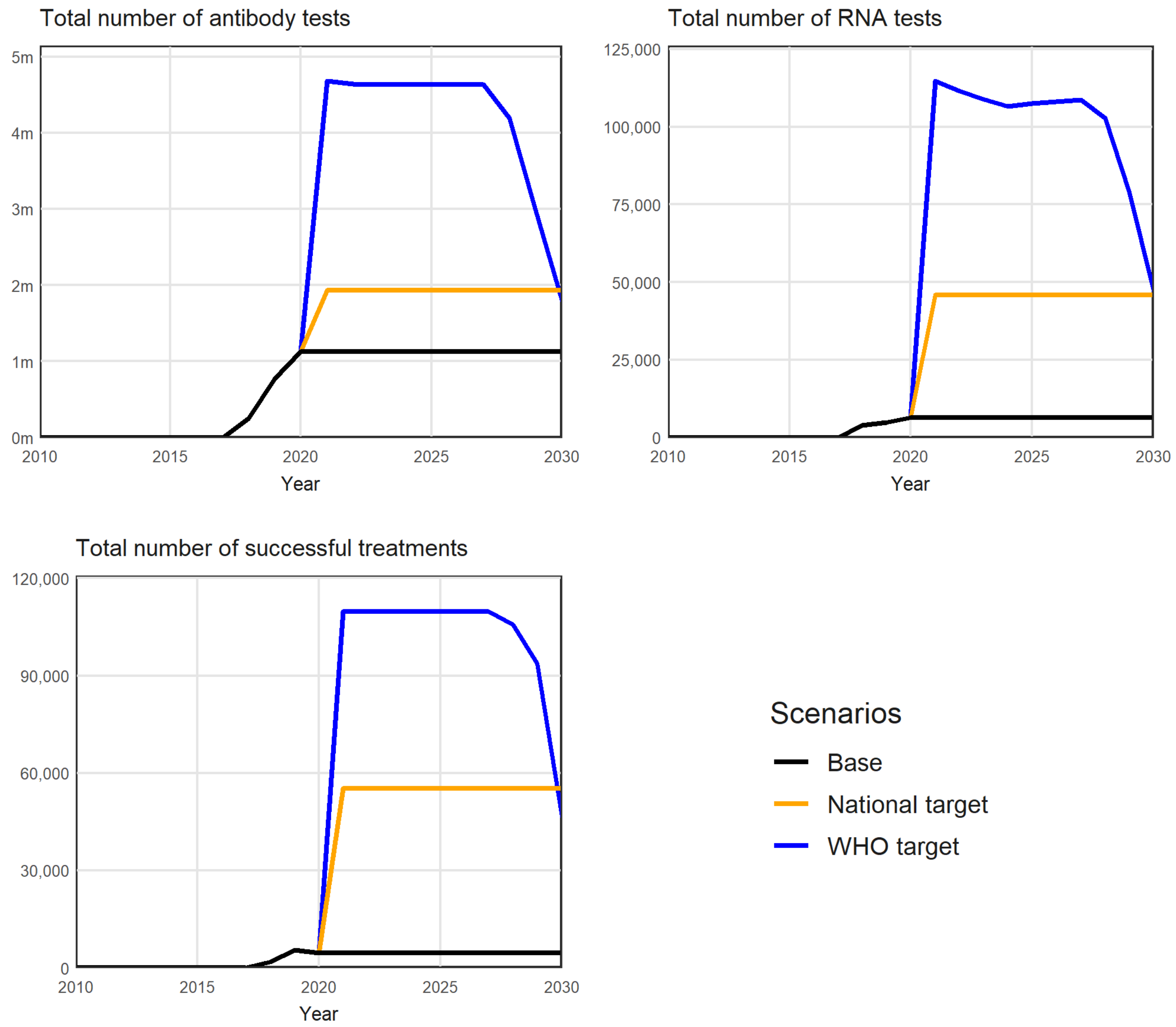

Figure S4: Estimated total number of tests and treatment courses between 2020 and 2030 required for each scenario.

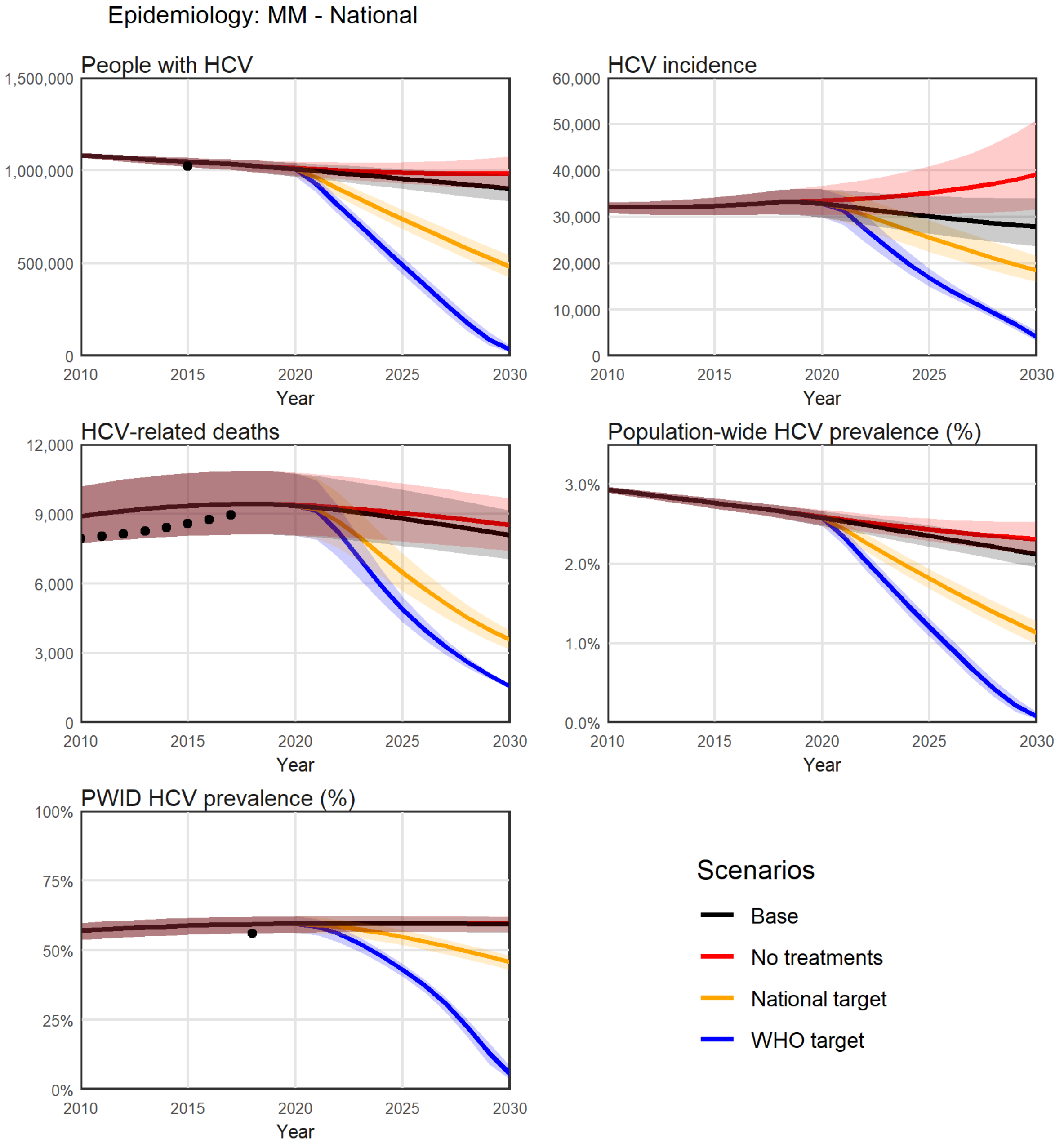

Figure S5: Epidemiological projections including a scenario of no testing or treatment. Projections for (A) people living with HCV; (B) HCV incidence; (C) HCV-related deaths; (D) prevalence of HCV in the general population; and (E) prevalence of HCV among people who inject drugs. Red line = no testing/treatment, black line = baseline, orange and blue lines = testing and treatment scaled up to reach the national strategy and WHO strategy targets respectively.

References

1. Scott N, McBryde E, Thompson A, Doyle J, Hellard M. Treatment scale-up to achieve global HCV incidence and mortality elimination targets: a cost-effectiveness model. *Gut* 2017; **66**(8): 1507-15.
2. Scott N, Doyle J, Wilson DP, et al. Reaching hepatitis C virus elimination targets requires health system interventions to enhance the care cascade. *International Journal of Drug Policy* 2017; **47**: 107-16.
3. Myanmar Information Management Unit. The 2014 Myanmar Population and Housing Census, 2016.
4. National AIDS Program, Ministry of Health and Sports Myanmar. Myanmar Integrated Biological and Behavioural Surveillance Survey & Population Size Estimates among People Who Inject Drugs (PWID) 2017-2018. Accessed 15 April 2019 from: https://www.aidsdatahub.org/sites/default/files/highlight-reference/document/Myanmar_IBBS_and_Population_size_estimates_among_PWID_2017-2018.pdf. 2019.
5. Aye Aye Lwin KSA, Moh Moh Htun, Yi Yi Kyaw, Ko Ko Zaw, Toe Thiri Aung, Myat Phone Kyaw, Khin Pyone Kyi, Kyaw Zin Thant. Sero-prevalence of Hepatitis B and C Viral Infections in Myanmar: National and Regional Survey in 2015. *Myanmar Health Sciences Research Journal, Vol 29, No 3, 2017* 2015.
6. Global Burden of Disease Collaborative Network. Global Burden of Disease Study 2017 (GBD 2017) Disability Weights. 2018. http://ghdx.healthdata.org/record/global-burden-disease-study-2017-gbd-2017-disability-weights.
