# Supplementary material for: Hepatitis C elimination in Myanmar: Modelling the impact, cost, cost-effectiveness and economic benefits
Source: Lancet Reg Health West Pac. 2021 Mar 23;10:100129. doi: 10.1016/j.lanwpc.2021.100129 (PMC8315611; doi:10.1016/j.lanwpc.2021.100129)
Supplement: Supplementary file 2 [file mmc2.docx]

**Supplementary material.** Appendix A, model description; Appendix B, summary of data inputs for the 15 states, regions and union territories of Myanmar; Appendix C, additional details of cost estimation; Appendix D, sub-national model projections; Appendix E, additional model outputs.
